# Visual Attention Deficits in Schizophrenia Can Arise From Inhibitory Dysfunction in Thalamus or Cortex

**DOI:** 10.1162/cpsy_a_00023

**Published:** 2018-12

**Authors:** Yohan J. John, Basilis Zikopoulos, Daniel Bullock, Helen Barbas

**Affiliations:** Neural Systems Laboratory, Department of Health Sciences, Boston University, Boston, Massachusetts, USA; 2 Human Systems Neuroscience Laboratory, Department of Health Sciences, Boston University, Boston, Massachusetts, USA; 3 Graduate Program for Neuroscience, Boston University, and School of Medicine, Boston, Massachusetts, USA; 4 Graduate Program for Neuroscience, Boston University, and School of Medicine, Boston, Massachusetts, USA; 5 Department of Psychological and Brain Sciences, Boston University, Boston, Massachusetts, USA; 6 Neural Systems Laboratory, Department of Health Sciences, Boston University, Boston, Massachusetts, USA; 7 Graduate Program for Neuroscience, Boston University, and School of Medicine, Boston, Massachusetts, USA

**Keywords:** psychiatry, thalamic reticular nucleus, computational model, spiking, GABA, NMDA, parvalbumin neurons, calbindin neurons, disinhibition

## Abstract

Schizophrenia is associated with diverse cognitive deficits, including disorders of attention-related oculomotor behavior. At the structural level, schizophrenia is associated with abnormal inhibitory control in the circuit linking cortex and thalamus. We developed a spiking neural network model that demonstrates how dysfunctional inhibition can degrade attentive gaze control. Our model revealed that perturbations of two functionally distinct classes of cortical inhibitory neurons, or of the inhibitory thalamic reticular nucleus, disrupted processing vital for sustained attention to a stimulus, leading to distractibility. Because perturbation at each circuit node led to comparable but qualitatively distinct disruptions in attentive tracking or fixation, our findings support the search for new eye movement metrics that may index distinct underlying neural defects. Moreover, because the cortico-thalamic circuit is a common motif across sensory, association, and motor systems, the model and extensions can be broadly applied to study normal function and the neural bases of other cognitive deficits in schizophrenia.

## INTRODUCTION

Schizophrenia affects large numbers of people worldwide and has been a focus of research for longer than a century (reviewed in Jablensky, 2000). Despite this interest, an integrated causal conception of the disorder has proven elusive. Several lines of evidence have linked schizophrenia with abnormalities in cortical interneurons that express GABA (Bastrup & Larsen, 2017; Beasley, Zhang, Patten, & Reynolds, 2002; Chung, Fish, & Lewis, 2016; Glausier & Lewis, 2017; Rotaru, Lewis, & Gonzalez-Burgos, 2012; Steullet et al., 2017; Woo, Shrestha, Lamb, Minns, & Benes, 2008), but the causal pathway from dysfunctional inhibition to disordered thought, emotion, and behavior remains unresolved. Computational modeling of neuronal networks provides an ideal tool to bridge the gap between neural correlates and high-level symptoms (Adams, Huys, & Roiser, 2015).

Instead of attempting to model the full spectrum of schizophrenia-related symptoms, it is more feasible and tractable in computational terms to probe representative symptoms manifested across patients in whom the expression of other symptoms may vary considerably. Attentive stimulus tracking represents a cluster of such common symptoms. For example, the ability to smoothly track a moving visual stimulus is degraded in schizophrenia patients. Oculomotor tracking is jerky rather than smooth and characterized by increased positional errors and more frequent “catch-up” saccades (Allen, Matsunaga, Hacisalihzade, & Stark, 1990; Benson et al., 2012; Clementz, Grove, Iacono, & Sweeney, 1992; Diefendorf & Dodge, 1908; Holzman, Proctor, & Hughes, 1973; Holzman et al., 1974; Iacono, Moreau, Beiser, Fleming, & Lin, 1992; Levy, Sereno, Gooding, & O’Driscoll, 2010; O’Driscoll & Callahan, 2008; Ross et al., 1997; Rybakowski & Borkowska, 2002). A recent study showed that a machine learning algorithm can use data from tests of oculomotor attention to distinguish schizophrenia patients from controls with a high degree of accuracy (Benson et al., 2012).

In addition to cortical inhibition, thalamic inhibition by the GABAergic thalamic reticular nucleus (TRN) has also been implicated in schizophrenia (Ferrarelli & Tononi, 2011, 2017; Pratt et al., 2017; Pratt & Morris, 2015). The connectivity pattern that integrates these two sources of inhibition is the cortico-reticulo-thalamic (CRT) circuit. The CRT circuit can participate in both normal and abnormal attention, as illustrated by our previous model of emotion-related regulation (John, Zikopoulos, Bullock, & Barbas, 2016). Here we focus on how the CRT circuit contributes to attention for stimulus tracking and how disruptions to specific sources of inhibition in the circuit can lead to abnormal performances observed in schizophrenia patients.

Our simulations showed that three sources of GABAergic inhibition may be crucial for normal stimulus tracking and attentive fixation: (a) a class of cortical parvalbumin-expressing (PV) interneurons (INs) that provide strong perisomatic inhibition of cortical pyramidal neurons (Freund & Katona, 2007), (b) a class of cortical calbindin-expressing (CB) INs that provide inhibition of the mid- to distal dendrites of cortical pyramidal neurons (DeFelipe, 1997; Kawaguchi & Kubota, 1997), and (c) TRN neurons, which provide inhibition to thalamic excitatory neurons that are connected with the cortex (reviewed in Zikopoulos & Barbas, 2007). The CB-IN class overlaps with the class of somatostatin (SOM) INs, because many of the mid- to distal inhibiting INs coexpress SOM and CB (González-Albo, Elston, & DeFelipe, 2001), and SOM interneurons are also affected in schizophrenia (Fung, Fillman, Webster, & Shannon Weickert, 2014; Guillozet-Bongaarts et al., 2014). In addition, we found that a recently discovered form of short-term plasticity, which transiently reduces inhibition of the thalamus by TRN (Crandall, Cruikshank, & Connors, 2015), may contribute to normal network performance. Isolated perturbations to any of these inhibitory systems yield some common symptoms but showed important differences in behavior and neural dynamics. The model supports the idea that schizophrenia-related symptoms can arise from distinct neural disorders. The differences between the disruptions may provide insights to unravel the mechanistic bases of distinct subtypes of schizophrenia.

## RESULTS

### Model Design: Key Model Elements, Connectivity, and Mechanisms

#### CRT Circuit

**Figure 1.**
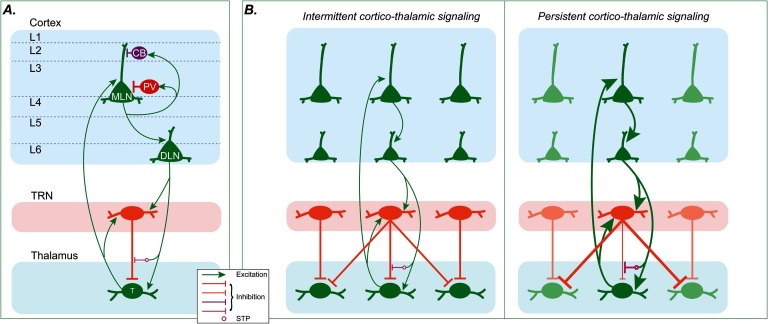
**Connectivity of the model.** A) Overall connectivity between neural groups. The CB-INs only inhibit the dendrites receiving distal inputs. The PV-INs inhibit the cell bodies of pyramidal neurons as well as each other. B) Disinhibitory short-term plasticity (dSTP). For clarity, local inhibitory INs in the cortex are omitted. Left: If the cortico-thalamic firing in a cortico-reticulo-thalamic (CRT) loop is weak or intermittent, closed-loop inhibition from thalamic reticular nucleus (TRN) is strong, preventing persistent thalamo-cortical signaling and facilitating global phasic or oscillatory firing modes. Right: If the cortico-thalamic firing in a CRT loop is strong and persistent, the dSTP mechanism causes closed-loop inhibition from TRN to be weakened, facilitating strong and focused thalamo-cortical signaling (dark red and green neurons) and a tonic firing mode. MLN = middle-layer neuron; DLN = deep-layer neuron; PV = parvalbumin interneuron; CB = calbindin interneruon; T = thalamo-cortical neuron.

The CRT circuit incorporates the main inhibitory systems in cortex and thalamus linked to schizophrenia and is therefore the focus of our computational model (Figure 1A). We modeled the known connectivity, which also formed part of our *emotional gatekeeper* (EmGate) model (John et al., 2016). The activity of each neuron is governed by simplified spiking dynamics (Izhikevich, 2003). The model cortex contains two groups of excitatory pyramidal neurons: middle-layer neurons (MLNs) and deep-layer neurons (DLNs).

The middle cortical layers also include two groups of inhibitory interneurons (INs): PV-INs and CB-INs (Figure 1A; Dombrowski, Hilgetag, & Barbas, 2001; Medalla & Barbas, 2006; Pouget et al., 2009). The PV- and CB-INs are each excited by nearby MLNs. The fast-spiking PV-INs provide feedback inhibition to nearby excitatory MLNs and to each other (DeFelipe, 1997; Freund & Katona, 2007). The CB-INs gate thalamo-cortical signaling by inhibiting the mid-distal dendrites of nearby MLNs (DeFelipe, 1997; Kawaguchi & Kubota, 1997). Dendritic inhibition of the type performed by the model CB-INs is also performed by somatostatin-expressing (SOM) interneurons (Dienel & Lewis, 2018; Scheyltjens & Arckens, 2016), which overlap with the CB-INs (González-Albo et al., 2001; Guillozet-Bongaarts et al., 2014). Thus our results pertain to SOM interneurons too. Several studies have also associated abnormalities in SOM-INs with schizophrenia (Fung et al., 2014; Guillozet-Bongaarts et al., 2014). High activity in CB-INs narrows the effective receptive fields of nearby excitatory MLNs. This type of function can cancel noise (Wang, Tegnér, Constantinidis, & Goldman-Rakic, 2004).

The thalamic neurons send topographic projections to the MLNs, which in turn send topographic projections to the DLNs. The DLNs send topographic projections to the dorsal thalamus and TRN, as described in classical and recent studies (Jones, 2007; Zikopoulos & Barbas, 2006). Figure 1B illustrates the connectivity between TRN and thalamus and the inhibitory interactions between parallel CRT loops. “Closed-loop” or “feedback” inhibition occurs within a CRT loop, meaning that a TRN neuron inhibits the thalamic neurons that excite it. “Open-loop” or “off-surround” inhibition is between CRT loops, meaning that a TRN neuron inhibits thalamic neurons belonging to parallel CRT loops. In the model, inhibitory projections from TRN to thalamus are all-to-all and therefore primarily open loop, though closed-loop connections are also present (Pinault, 2004; Willis, Slater, Gribkova, & Llano, 2015).

Gap junctions linking adjacent TRN neurons facilitate synchronized TRN activity (Steriade, 2005). Model neurons in TRN and thalamus also express low-threshold T-type calcium channels (Destexhe, Contreras, Steriade, Sejnowski, & Huguenard, 1996), which allow for oscillations in the alpha and delta ranges, driven by hyperpolarization (Destexhe et al., 1996; Steriade, McCormick, & Sejnowski, 1993; Vijayan & Kopell, 2012). Reduced suppression of theta-alpha oscillations during sensory gating has been associated with schizophrenia (Hong et al., 2008).

#### Disinhibitory Short-Term Plasticity

In the open-loop connectivity pattern of the CRT circuit, most inhibitory presynaptic synapses on a given thalamic neuron originate in TRN neurons belonging to parallel CRT loops, and only a minority originate in TRN neurons that receive reciprocal excitation from targeted thalamic neurons. This connectivity enables lateral inhibition between parallel CRT loops as well as negative feedback within a CRT loop.

The strength of negative feedback is modulated by disinhibitory short-term plasticity (dSTP) of the synapses from TRN to thalamus, controlled by activity in the deep layers of cortex (Crandall et al., 2015). This cortex-mediated disinhibition occurs alongside a weaker short-term facilitation by cortical layer 6 projection to the dorsal thalamus. The latter is both small and synergistic with the reduction in thalamic inhibition (Crandall et al., 2015). Preliminary simulations (not shown) indicated that inclusion of the cortical facilitation on the dorsal thalamus does not alter the qualitative performance of the model, and thus we did not include it.

The dSTP mechanism, when combined with focal, topographic projections from cortex to thalamus, allows creation of specific, targeted “islands” of reduced TRN inhibition. If a given DLN maintains sustained high activity (for approximately 200 ms or longer), the corresponding thalamic neurons undergo weakened inhibition from the active TRN neurons. In the absence of such islands of reduced inhibition, all neurons in the thalamus receive the same level of inhibition from each TRN neuron firing at a given rate. Figure 1B depicts how the dSTP mechanism affects thalamo-cortical signaling in the model.

The dSTP mechanism is triggered by persistent elevated activity in the cortical DLNs (Crandall et al., 2015) and is implicated in the ability to sustain attention to meet task requirements (Sparks et al., 2018; Wimmer et al., 2015). The mechanism enables disinhibition of a localized region of thalamus whenever there is strong and topographically invariant firing in the corresponding DLNs. In other words, persistent firing in a set of cortico-thalamic neurons disinhibits the corresponding thalamo-cortical neurons, enabling them to respond more strongly to their inputs and therefore to communicate more robustly with their targets in cortex and elsewhere in the brain. We therefore refer to this mechanism as *selective disinhibition*. It can be understood as a form of top-down priming that does not involve excitation of neurons representing an expected stimulus. This form of indirect priming has the benefit of avoiding top-down excitation of thalamic neurons in the absence of bottom-up input. If direct excitatory priming is excessively high, the signals sent by thalamic neurons to the cortex and elsewhere will incorrectly convey the presence of bottom-up input even when absent.

#### Perturbing Cortical and TRN Inhibitory Neurons

Dysfunction of glutamatergic NMDA receptors on cortical PV- and CB-INs has been linked to schizophrenia (Alherz, Alherz, & Almusawi, 2017; Coyle, 2004; Kehrer, Maziashvili, Dugladze, & Gloveli, 2008; Olney, Newcomer, & Farber, 1999; Olney & Farber, 1995). In our simulations, we thus perturbed cortical INs by reducing their excitation by glutamatergic inputs. Specifically, we decreased the conductances of the NMDA receptors on the INs. Reduced perisomatic inhibition from PV-INs results in higher firing rates in MLNs, whereas reduced dendritic inhibition from CB-INs broadens the receptive fields of MLNs.

Perturbation through direct reduction of GABA in PV-INs is likely to be less subtle than for NMDA receptors. Deficiency in GABA in the brains of schizophrenia patients has generally been demonstrated by expression of its biosynthetic enzymes (e.g., Volk et al., 2012). A recent study showed that even a small reduction (14%–16%) of the biosynthetic enzyme GAD67 in GABAergic boutons in prefrontal cortex is sufficient to differentiate the brains of schizophrenia patients from controls (e.g., Rocco, Lewis, & Fish, 2016). Supplemental simulations revealed that direct reduction of the strength of GABA had a broadly similar effect, because the NMDA conductance decrease and the direct decrease of GABA could both reduce the strength of inhibition onto the MLNs. The effectiveness of the NMDA perturbation requires that the NMDA conductance contribute appreciably to the firing rate of the INs.

We did not separately examine a deficit of NMDA receptors on pyramidal neurons. If pyramids also suffer from NMDA dysfunction, available data suggest that such a dysfunction would lead to increased network activity (Kimoto et al., 2016; Tatard-Leitman et al., 2015) and hence synergize with, that is, amplify, the NMDA deficit of PV-INs. Therefore the simulations with a strong PV-IN deficit, or direct reduction of GABA release, already pertain to this case.

We perturbed the TRN neurons through direct hyperpolarization, which led to deinactivation of their T-type calcium channels. The opening of these channels puts the TRN in a phasic mode consisting of periodic bursts of activity (Destexhe et al., 1996). Last, we perturbed the dSTP mechanism by reducing the strength of the selective disinhibition of thalamus that normally emerges from persistent DLN activation.

#### Error-Guided Tracking and Fixation

The model enables stimulus tracking and fixation through a phenomenological mechanism: the pattern of activity in the cortical MLNs is treated as a retinal eccentricity map that drives horizontal corrective eye movements. Thalamic inputs arrive in retinotopic coordinates, so the “left-side neurons” receive inputs from the left of the retinal image and the “right-side neurons” receive input from the right side of the image. Activations of the left-side neurons combine additively to produce leftward movement of the model’s “eye,” and the activities of the right-side neurons produce rightward movement. Thus, when a stimulus is left of center, the field of view shifts to the left, and when it is right of center, the field of view shifts to the right. When the system functions correctly, activity in the network is relatively stable because the target stimulus is maintained in the center of view. As the retinal eccentricity or error representation is used to generate corrective movements, we refer to it as a *motor error map*. Here *error* refers to retinal position and not error signals that might occur at the time of an expected reinforcement.

The model is set up so that its intrinsic goal is foveation of the target. Whenever it detects a nonfoveated target on its internal map, it automatically treats the event as an error. Excitation outside the foveal region of the map leads to corrective motor commands that adjust the gaze angle to reduce the eccentricity of the target’s location relative to the foveal part of the map. Thus the term *motor error map* is shorthand for “neural map mediating the sensory-motor transform that corrects nonzero retinal eccentricity of the target stimulus.” The model does not include prediction of target position or velocity and therefore relies solely on error-guided input to drive eye movements. In moments when the error is exactly zero, leftward and rightward movement signals are symmetrical and therefore counterbalanced. Following foveation of a yet-moving target, the error soon becomes nonzero again, because there is no target-velocity signal that could cause gaze velocity to match target velocity. In the case of fixation on a stationary target, noise may break the symmetry of error map outputs, and if this occurs, the system will generate small back-and-forth movements across the target. Such oscillatory movement is a necessary consequence of purely error-driven tracking, provided that signal integration is not instantaneous and that error-correcting signals are strong enough to fully zero the gaze error.

There are several ways to disrupt the normal functioning of the error map. If the number of active neurons in the error map is constant, then increasing or decreasing the firing rates will cause excessively large or small movements, respectively. Conversely, the number of active neurons in the error map can change while their individual firing rates remain roughly constant. This can lead to a potentially counterintuitive effect: higher recruitment of error map neurons alongside slower movements. Such a situation arises if the movement-generating system receives strong but conflicting signals that direct the eye to both sides simultaneously. A large leftward signal may be accompanied by a smaller rightward signal, resulting in a lagged leftward movement. Distractor stimuli also disrupt the error map by triggering irrelevant error signals that lead to extraneous movements. Thus the attentional system and the motor error map influence each other, creating a perception–action loop.

#### Circuit for Oculomotor Behavior

The model does not specify most of the numerous brain structures associated with oculomotor behavior. The single CRT loop modeled here merges the functional roles of several parallel, interacting CRT loops. Subcortical structures, including the superior colliculus (SC), are also likely involved. Several lines of evidence suggest that the CRT loop linking the frontal eye field (FEF, Brodmann area 8), TRN, and medial dorsal thalamus (MD) is a prime candidate for involvement in the schizophrenia-related disruptions modeled here (reviewed in Schall, 2015). The FEF is known to possess retinotopic receptive fields (Caruso, Pages, Sommer, & Groh, 2018), which can be interpreted as retinal eccentricity maps (Schall, 2015).

In addition to the folding together of target recognition and the mapping between retinal eccentricity and eye movement commands, the model introduces a functional simplification pertaining to the representation of the task structure. Instead of an explicit task instruction signal, the model employs a mechanism to mediate “task-on” behavior. In humans, explicit instructions are required to induce subjects to track a moving stimulus or ignore distractors. Without such instructions, normal subjects are susceptible to attentional capture by distractors and novelty. Here we do not model explicit task instructions, so the network is set up to be in a task-on mode.

The circuit modeled here is thus a simplification of the distributed network involved in the full range of visual spatial attention and tracking. Detailed computational models (e.g., Srihasam, Bullock, & Grossberg, 2009) incorporate additional regions required for processes like voluntary target selection (Krauzlis, Lovejoy, & Zénon, 2013; Rao, Mayo, & Sommer, 2016; Smith, Cotton, Bruno, & Moutsiana, 2009; Tian & Lynch, 1997). Here we abstracted and simplified the diverse visuomotor behaviors to focus on an anatomical circuit that can mediate basic stimulus tracking and selective attention and whose function can be generalized to other systems that incorporate the CRT circuit motif. We explore how the model relates to the wider visuomotor circuit in the Discussion.

### Simulations: Normal Smooth Stimulus Tracking and Attentional Fixation

We modeled the CRT circuit (Figure 1A) as a network of spiking neurons (Izhikevich, 2003) and tested its performance on two behavioral tasks that require sustained attention: smooth tracking of a moving target and fixation on a stationary target despite presentations of a distractor; both behaviors are impaired in schizophrenia (Benson et al., 2012). Our simulations revealed that the CRT circuit enables smooth stimulus tracking as well as attentional fixation, both of which can be disrupted by manipulations of key inhibitory processes. We thus infer that inhibitory neurons in cortex and thalamus contribute significantly to normal stimulus tracking and attention.

In the two simulated tasks, both target and distractor are Gaussian bumps in the rate of the Poisson input to the thalamus. In the smooth tracking task, the center of the target moves in a sinusoidal pattern along the horizontal with a frequency of 1 Hz. In the fixation task, a stationary target is presented first, and then a stationary distractor of equal width and intensity is introduced at the 2 s mark at a different horizontal locus. In each simulation, after an initial period of oscillatory gaze-error reduction, the model converges to keep the target stimulus centered in retinotopic space. This period varies depending on input noise. We do not explicitly model the neural correlates of a task instruction or a goal state. The model therefore converges on accurate stimulus tracking as the natural or default state. The final quarter of each trial is the perturbation epoch.

#### Smooth Pursuit of a Moving Stimulus

**Figure 2.**
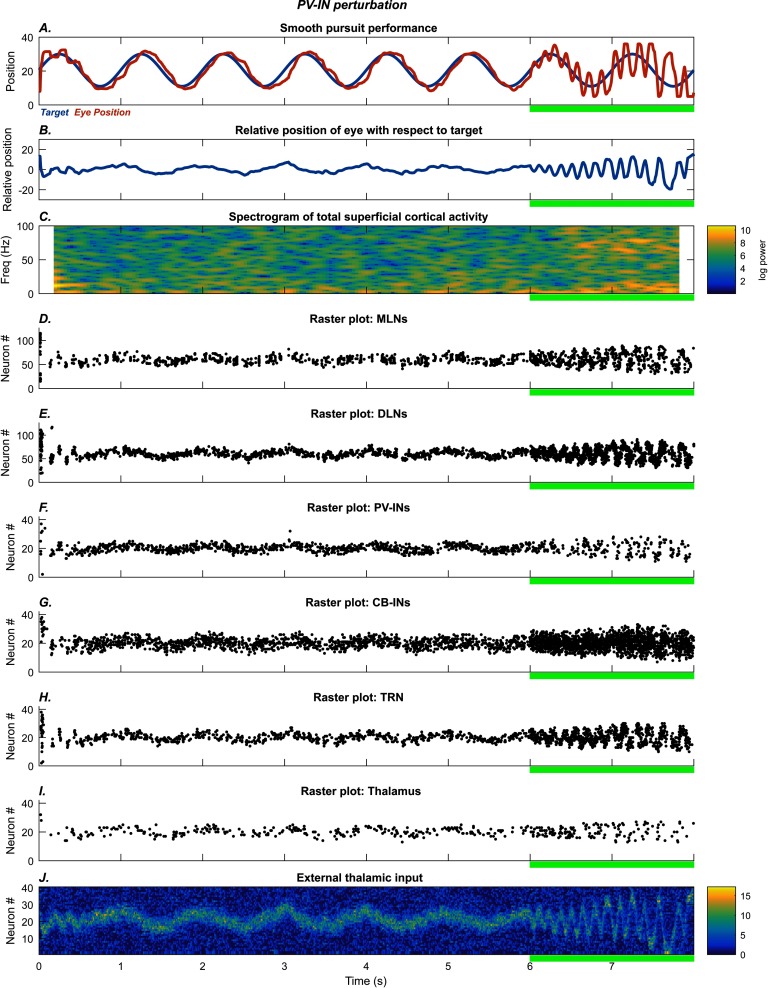
**PV-IN perturbation: smooth pursuit task.** The system quickly settles on accurate smooth pursuit of the stimulus. After the 6 s mark (green bar), glutamatergic NMDA conductance on the PV-INs is reduced, leading to increased variability in eye movement. The subplots show the time evolution of model behavior (A–B) and model neural activities (C–I) as well as external thalamic input (J). A) Horizontal position of the model eye (red trace) and the target (blue trace) in absolute coordinates. B) Relative position of the eye with respect to the target. C) Spectrogram of total middle-layer cortical activity. D–I) Raster plots show spiking activity in the model neurons. J) External thalamic input.

Figure 2 shows behavioral performance (Figures 2A and 2B) and neural activity patterns (Figures 2C–2I) in the smooth tracking task. After a brief period of gaze-error reduction, the model’s gaze direction, hereinafter *eye position* (Figure 2A, red trace), became aligned with that of the target (Figure 2A, blue trace), so that residual error in eye position fluctuated near zero (Figure 2B). Prior to convergence on accurate performance, cortical MLN activity was in an alpha oscillatory mode (∼10 Hz), as shown in the spectrogram (Figure 2C). The alpha mode reduced once gaze direction became accurate, within the first second of the trial. During this alpha epoch, closed-loop inhibition from TRN to thalamus occurred, so the thalamus “sampled” the external stimulus pattern intermittently. This kind of intermittent mode of stimulus selection will be referred to as *intermittent attention*. Figures 2D–2I show spike rasters of the neuron groups, each of which is retinotopic. A centered activity pattern therefore corresponds to zero displacement of the target, or zero error. The cortical MLNs (Figure 2D) serve as a motor error map: When activity is greater in the right half of the neural group, movement is to the right, and when activity is greater in the left half, movement is to the left.

The cortical MLNs excite the cortical DLNs (Figure 2E), which in turn send topographic projections to TRN (Figure 2H) and the thalamus (Figure 2I). The MLNs also excite the cortical PV-INs (Figure 2F) and the cortical CB-INs (Figure 2G) in a topographic manner. Each PV-IN provides nearby MLNs with strong inhibition. Each CB-IN provides nearby MLNs with inhibition on the distal dendrites, effectively regulating the width of the receptive field of each MLN. Strong CB-IN firing causes the corresponding MLNs to respond to a narrower band of thalamic neurons. Sustained activity in the DLNs triggers dSTP of the inhibition from TRN to the corresponding thalamic neurons, resulting in *selective disinhibition*. This sustained firing is reflected in the spike rasters prior to the 6 s point (Figures 2D–2I). When selective disinhibition rose to its highest possible level, the disinhibited thalamic neurons (Figure 2I) became maximally responsive to their inputs (Figure 2J), enabling the MLNs to receive more continuous and strong stimulus information. In other words, activity in DLNs created “islands” of thalamic disinhibition. One consequence of the selective disinhibition mediated by the cortex was to shift the model from intermittent attention to continuous attention. In intermittent attention, no CRT loop remains strong enough to suppress competitors for very long. In this condition, attention is spread widely but thinly. In contrast, during continuous attention, the CRT loop with the most active DLNs successfully suppresses competitors.

#### Fixation on a Stationary Target in the Presence of a Distractor

**Figure 3.**
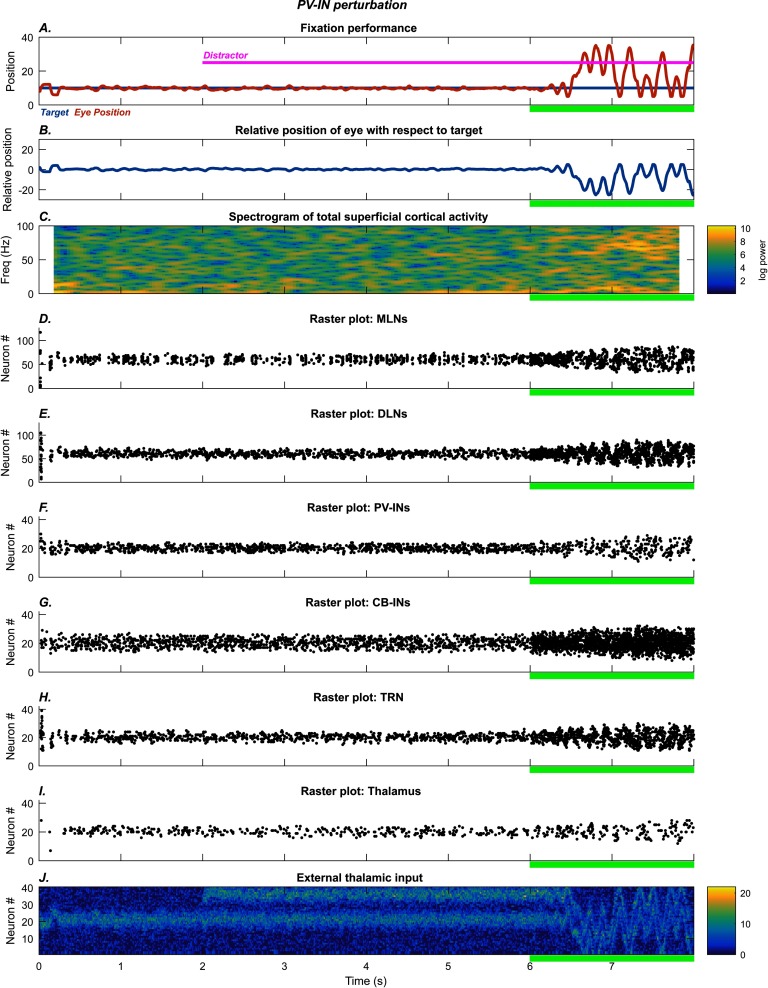
**PV-INs perturbation: fixation task.** The system quickly settles on accurate fixation of the stationary stimulus. A distractor (magenta trace) is introduced at the 2 s mark, but fixation performance is unaffected. After the 6 s mark (green bar), glutamatergic NMDA conductance on the PV-INs is reduced, causing the model to shift between target and distractor. The subplots show the time evolution of model behavior (A–B) and model neural activities (C–I) as well as external input (J). A) Horizontal position of the model eye (red trace), the target (blue trace), and the distractor (magenta trace, straight line) in absolute coordinates. B) Relative position of the eye with respect to the target. C) Spectrogram of total middle-layer cortical activity. D–I) Raster plots show spiking activity in the model neurons. J) External thalamic input.

Figure 3 shows behavioral performance (Figures 3A and 3B) and neural activity patterns (Figures 3C–3I) in the attentional fixation task. After a brief period in the intermittent attention mode, the system stabilized so that the model eye (Figure 3A, red trace) aligned with the stationary target (Figure 3A, blue trace). Even when the distractor stimulus was turned on at the 2 s mark (Figure 3A, magenta trace), the average error in eye position (Figure 3B) oscillated around zero, implying that the distractor was ignored. As in the smooth pursuit task, this occurred because of sustained activity in the cortical DLNs, which triggered dSTP of the TRN inhibition on the thalamus.

### Simulation of Specific Perturbations in the CRT Loop

#### Role of Cortical PV Interneurons

To investigate how disruption of cortical PV-INs affects performance, we reduced the NMDA conductance on PV-INs by 90% during the final two seconds of each task. After the 6 s mark, the NMDA input to the PV-INs (Figures 2F and 3F) was diminished, leading to weaker inhibition of the cortical MLNs (Figures 2D and 3D). This resulted in greater activity in the MLNs, which serve as an error map, leading to larger amplitude eye movements. In the smooth pursuit task, the net effect was periodic overshoot of the target position (Figure 2A). While this effect originated in the cortex, it produced synergistic effects in the thalamus. The oscillatory eye movement caused a wider region of the MLN group to be active, which then caused a wider region of the DLN group to be active (Figures 2E and 3E). Thus the region of the thalamus undergoing selective disinhibition increased, allowing for increased susceptibility to distractors, seen most clearly in the fixation task, where the model eye moved between target and distractor after the PV-INs were disrupted (Figure 3A). This widening of the island of selective disinhibition effectively increased the size of the region for capture of attention by stimuli.

#### Role of Cortical CB Interneurons

**Figure 4.**
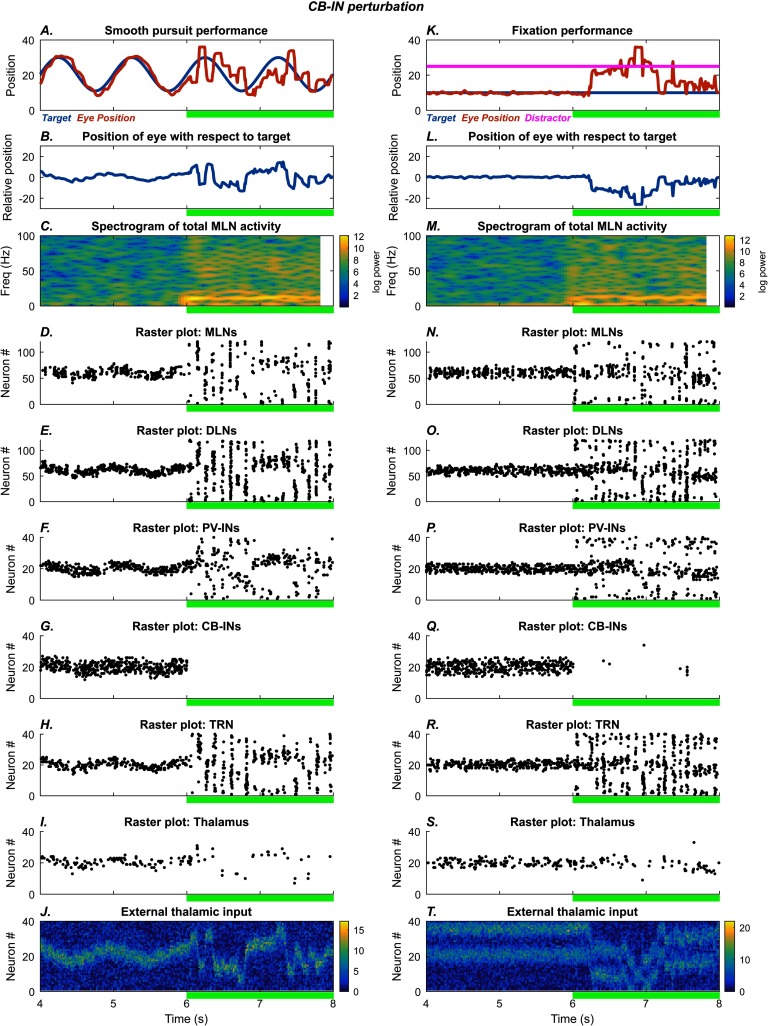
**CB-IN perturbation.** Left: Smooth pursuit task: final 4 s. After the 6 s mark (green bar), glutamatergic NMDA conductance on the CB-INs is reduced, leading to increased variability and lag in eye movement. The subplots show the time evolution of model behavior (A–B) and model neural activities (C–I) as well as external input (J). A) Horizontal position of the model eye (red trace) and the target (blue trace) in absolute coordinates. B) Relative position of the eye with respect to the target. C) Spectrogram of total middle-layer cortical activity. D–I) Raster plots show spiking activity in the model neurons. J) External thalamic input. Right: Fixation task: final 4 s. The system settles on accurate fixation of the stationary stimulus. A distractor (magenta trace, straight line) is introduced at the 2 s mark, but fixation performance is unaffected. After the 6 s mark (green bar), glutamatergic NMDA conductance on the CB-INs is reduced, causing the model to shift between target and distractor. The subplots show the time evolution of model behavior (K–L) and model neural activities (M–S) as well as external input (T). K) Horizontal position of the model eye (red trace), the target (blue trace), and the distractor (magenta trace) in absolute coordinates. L) Relative position of the eye with respect to the target. M) Spectrogram of total middle-layer cortical activity. N–S) Raster plots show spiking activity in the model neurons. T) External thalamic input.

Figure 4 shows the effects of disrupting cortical CB-INs on the smooth pursuit (left) and fixation (right) tasks, respectively. Reduction of NMDA conductance on CB-INs by 90% after the 6 s mark (Figures 4G and 4Q) led to negligible inhibition of the distal dendrites of the MLNs (Figures 4D and 4N) and widened the receptive field of each MLN. In turn, this caused an increase in synchronization among the MLNs, as the input patterns for the MLNs became more similar to each other. The synchronization is reflected in an increase in the power of the alpha band for cortical MLN activity (Figures 4C and 4M) and can also be discerned in the time domain in the spike rasters (Figures 4D and 4N). Owing to the increased number of active MLNs, broadband increases in power are also visible in the spectrogram. The increased number of active DLNs led to greater recruitment of TRN neurons (Figures 4H and 4R), causing broader thalamic inhibition and the elevated alpha band oscillation. The timescales across the CRT circuit are such that only synchronized TRN recruitment reliably creates the alpha rhythm. The net effect of the CB-IN disruption was more lagged movement in the smooth pursuit task (Figure 4A), that is, greater hysteresis, and more susceptibility to the distractor in the fixation task (Figure 4K). In both tasks, eye movements also became more erratic and variable.

#### Role of TRN Neurons

**Figure 5.**
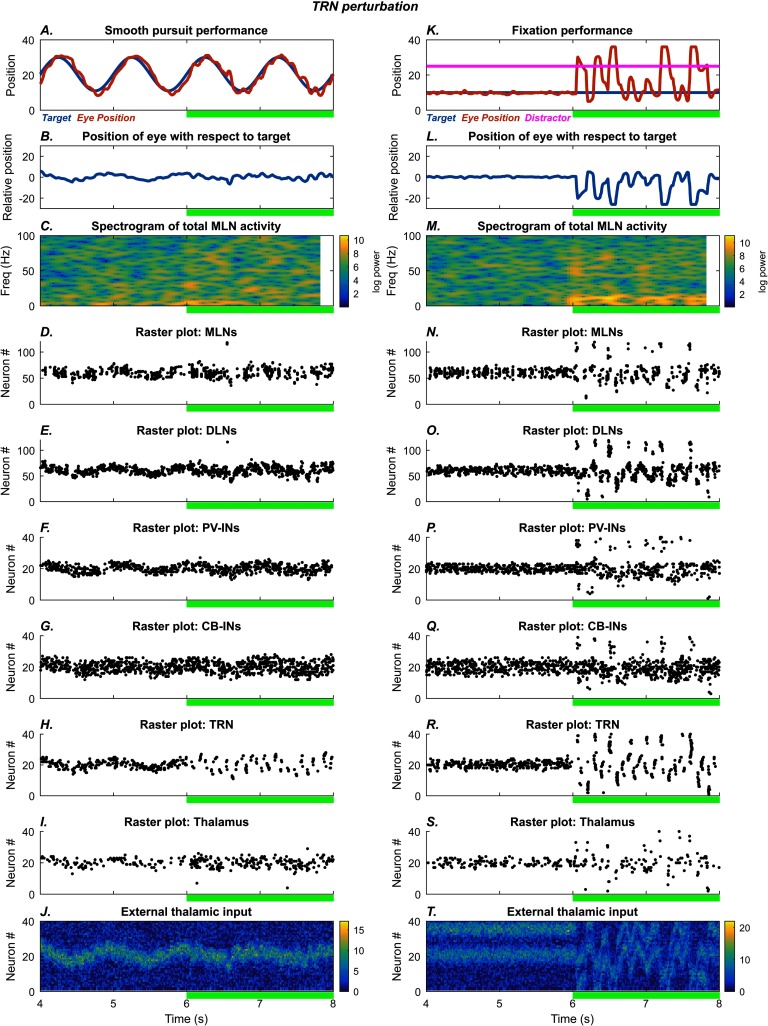
**TRN perturbation.** Left: Smooth pursuit task: final 4 s. After the 6 s mark (green bar), TRN neurons are hyperpolarized, leading to increased variability in eye movement. The subplots show the time evolution of model behavior (A–B) and model neural activities (C–I) as well as external input (J). A) Horizontal position of the model eye (red trace) and the target (blue trace) in absolute coordinates. B) Relative position of the eye with respect to the target. C) Spectrogram of total middle-layer cortical activity. D–I) Raster plots show spiking activity in the model neurons. J) External thalamic input. Right: Fixation task: final 4 s. The system settles on accurate fixation of the stationary stimulus. A distractor (magenta trace, straight line) is introduced at the 2 s mark, but fixation performance is unaffected. After the 6 s mark (green bar), TRN neurons are hyperpolarized, causing the model to shift between target and distractor. The subplots show the time evolution of model behavior (K–L) and model neural activities (M–S) as well as external input (T). K) Horizontal position of the model eye (red trace), the target (blue trace), and the distractor (magenta trace) in absolute coordinates. L) Relative position of the eye with respect to the target. M) Spectrogram of total middle-layer cortical activity. N–S) Raster plots show spiking activity in the model neurons. T) External thalamic input.

Figure 5 shows the effects of disrupting TRN inhibition on the smooth pursuit and fixation tasks. After the 6 s mark, TRN neurons (Figures 5H and 5R) were hyperpolarized sufficiently to activate low-threshold calcium channels. Consequently, rebound bursts of activation recurred periodically in the alpha range, causing intermittent bursts of inhibition on the thalamus and epochs of cortical oscillation in the alpha range and below (Figures 5C and 5M). As in the PV-IN simulations, the oscillatory eye movements occurred in the smooth pursuit task (Figure 5A), and there was increased susceptibility to noise and attention capture by distractors (Figure 5K).

#### Role of “Selective Disinhibition”

**Figure 6.**
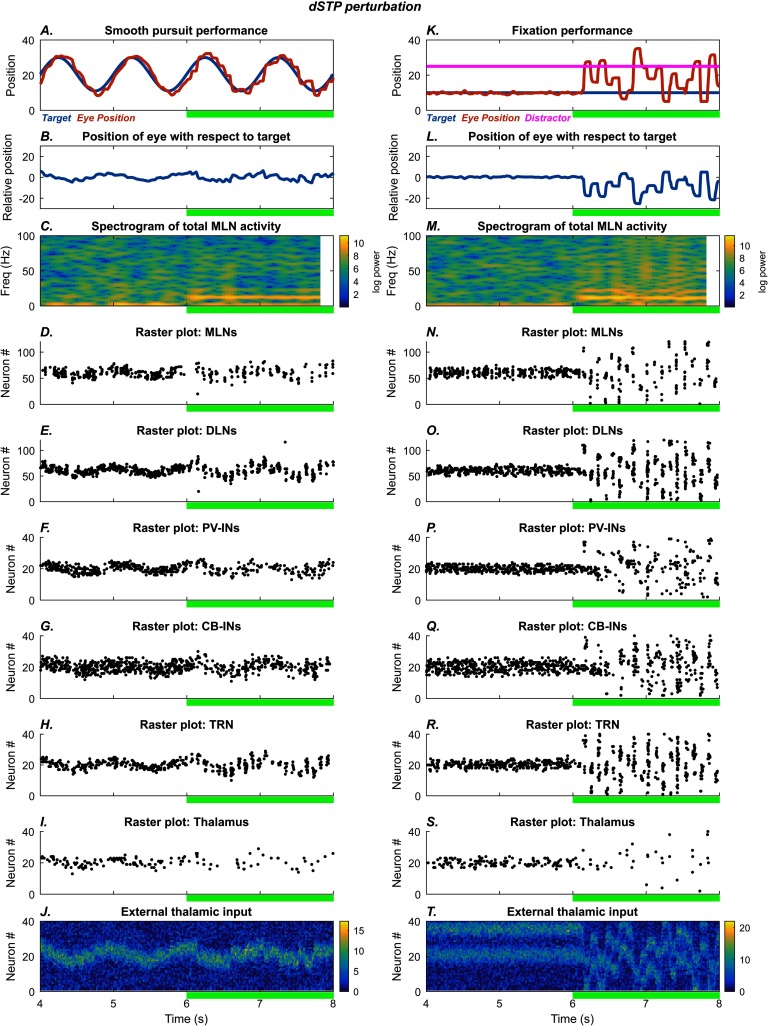
**dSTP perturbation.** Left: Smooth pursuit task: final 4 s. After the 6 s mark (green bar), the disinhibitory short-term plasticity (dSTP) of reticulo-thalamic inhibition is shut off, leading to increased variability in eye movement. The subplots show the time evolution of model behavior (A–B) and model neural activities (C–I) as well as external input (J). A) Horizontal position of the model eye (red trace) and the target (blue trace) in absolute coordinates. B) Relative position of the eye with respect to the target. C) Spectrogram of total middle-layer cortical activity. D–I) Raster plots show spiking activity in the model neurons. J) External thalamic input. Right: Fixation: final 4 s. The system settles on accurate fixation of the stationary stimulus. A distractor (magenta trace, straight line) is introduced at the 2 s mark, but fixation performance is unaffected. After the 6 s mark (green bar), the dSTP regulation reticulo-thalamic inhibition is shut off, causing the model to shift between the target and the distractor. The subplots show the time evolution of model behavior (K–L) and model neural activities (M–S) as well as external input (T). K) Horizontal position of the model eye (red trace), the target (blue trace), and the distractor (magenta trace) in absolute coordinates. L) Relative position of the eye with respect to the target. M) Spectrogram of total middle-layer cortical activity. N–S) Raster plots show spiking activity in the model neurons. T) External thalamic input.

When the cortical DLNs are strongly and persistently active, they activate dSTP, which reduces the effect of TRN inhibition on the thalamus. Through this novel mechanism, cortex has the ability to “preselect” specific regions of the thalamus by disinhibiting them. The cortex can thus create targeted islands of disinhibition in the thalamus that can communicate more strongly with cortex if they receive input. Figure 6 shows the effects of reducing the strength of dSTP by 90% on the smooth pursuit and fixation tasks. In both tasks, the lack of selective disinhibition after the 6 s mark put the system back in the intermittent attention mode, resulting in alpha oscillations, along with some increase in power in higher frequency bands, especially for the fixation task (Figures 6C and 6M). The cortical MLNs received a weaker but more widespread pattern of excitation from the thalamus, which caused erratic eye movements and increased susceptibility to noise and capture of attention by distractors (Figures 6A and 6K).

#### Similarities and Differences Among the Simulated Perturbations

**Figure 7.**
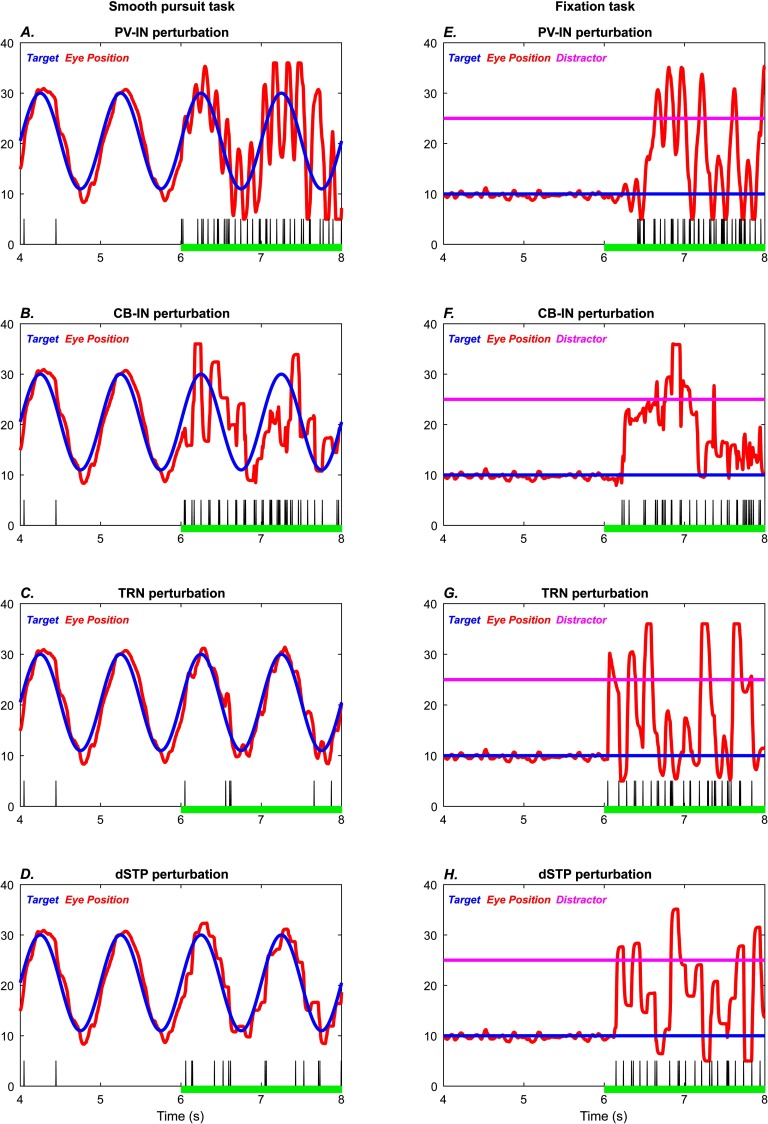
**Comparison of eye movements across perturbations.** Behavioral performance for samples of each type of simulated perturbation, zooming in on the final 4 s of each simulation. The plots on the left (A–D) show the smooth pursuit task, and the plots on the right (E–H) show the fixation task. Blue traces, target; red traces, eye position; magenta traces (straight lines), distractor. Vertical black tick marks indicate temporal locations of “saccade-like” high-velocity movement bursts. The perturbations of PV-INs (A, E), CB-INs (B, F), TRN neurons (C, G), and short-term plasticity (D, H) take place from the 6 s mark onward (green bar). They show broad qualitative similarities, but the detailed performance profiles show differences.

All perturbations resulted in disruptions to smooth pursuit and attentional fixation. More specifically, they led to rapid small-amplitude eye movements with occasional large-amplitude eye movements that resemble catch-up saccades, as well as attention capture by distractors. But the movement patterns also reveal key qualitative differences (subplots A and B of Figures 3–6, summarized in Figure 7). The PV-IN manipulation caused oscillatory eye movements: In the smooth pursuit task (Figure 7A), tracking was roughly accurate on average. In the fixation task, however, PV perturbation led to both erratic fixation and susceptibility to the distractor (Figure 7E). The CB-IN manipulation, by contrast, involved more discrete eye movements and more lagged performance in the smooth pursuit task (Figure 7B). The TRN manipulation led to small oscillatory disruptions in the smooth pursuit task (Figure 7C), which resembled the effect of the PV-IN perturbation, with very sharp and discrete saccade-like movements in the fixation task (Figure 7G). Similarly, the dSTP manipulation had a less pronounced effect on the smooth pursuit task (Figure 7D) than on the fixation task (Figure 7H). In each plot, the vertical black ticks indicate the temporal locations of high-velocity movement bursts, which are used in the quantitative plots discussed in what follows.

**Figure 8.**
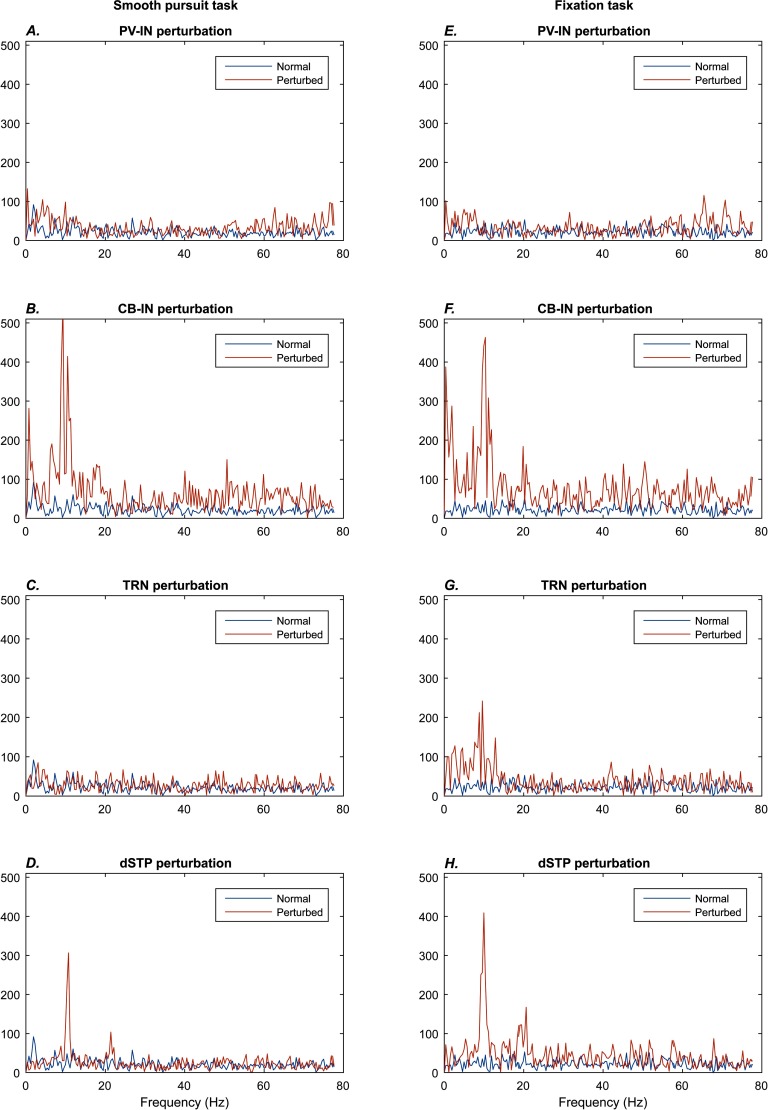
**Comparison of rhythmic activity across perturbations.** Each plot shows the power spectrum of the Fourier transform for the 2 s perturbation epoch (red traces) and the 2 s period immediately before the perturbation (blue traces). The plots on the left (A–D) show the smooth pursuit task, and the plots on the right (E–H) show the fixation task. In the perturbations of PV-INs (A, E), there is no evidence of change in alpha power. The perturbations of CB-INs (B, F) and short-term plasticity (D, H) show clear increases in the alpha band for both tasks. The TRN perturbation only shows an increase in alpha power in the fixation task (G).

The qualitative differences in performance are reflected in differences at the level of neural dynamics. The PV-IN perturbation caused oscillatory eye movements of roughly 10 Hz frequency (Figures 2A and 3A), but this oscillation was not strongly reflected in the spectrogram of MLN activity (Figures 2C and 3C; Figures 8A and 8E). Some epochs of increase in higher bands (high beta to gamma) also occurred for the PV-IN perturbation, due to disinhibition of MLNs (Figures 2C and 3C). By contrast, the CB-IN perturbation was accompanied by an increase in alpha power (Figures 4C and 4M; Figures 8B and 8F), reflected in the activity patterns of all the neuron groups (Figures 4D–4I and 4N–4S). Broadband increases in power also occurred due to increased number of active MLNs. The TRN perturbation showed epochs of increased power in the alpha band and in slower frequency bands (Figures 5C and 5M; Figures 8C and 8G) and also slower traveling waves in TRN, especially for the fixation task (Figures 5C and 5M; Figures 8C and 8G). The dSTP perturbation also caused increases in alpha power (Figures 6C and 6M; Figures 8D and 8H). The CB-IN, TRN, and dSTP manipulations also led to small increases in power in broad bands in higher frequency ranges (high beta to gamma), which primarily arise due to wider recruitment of MLNs.

#### Additional Perturbations

**Figure 9.**
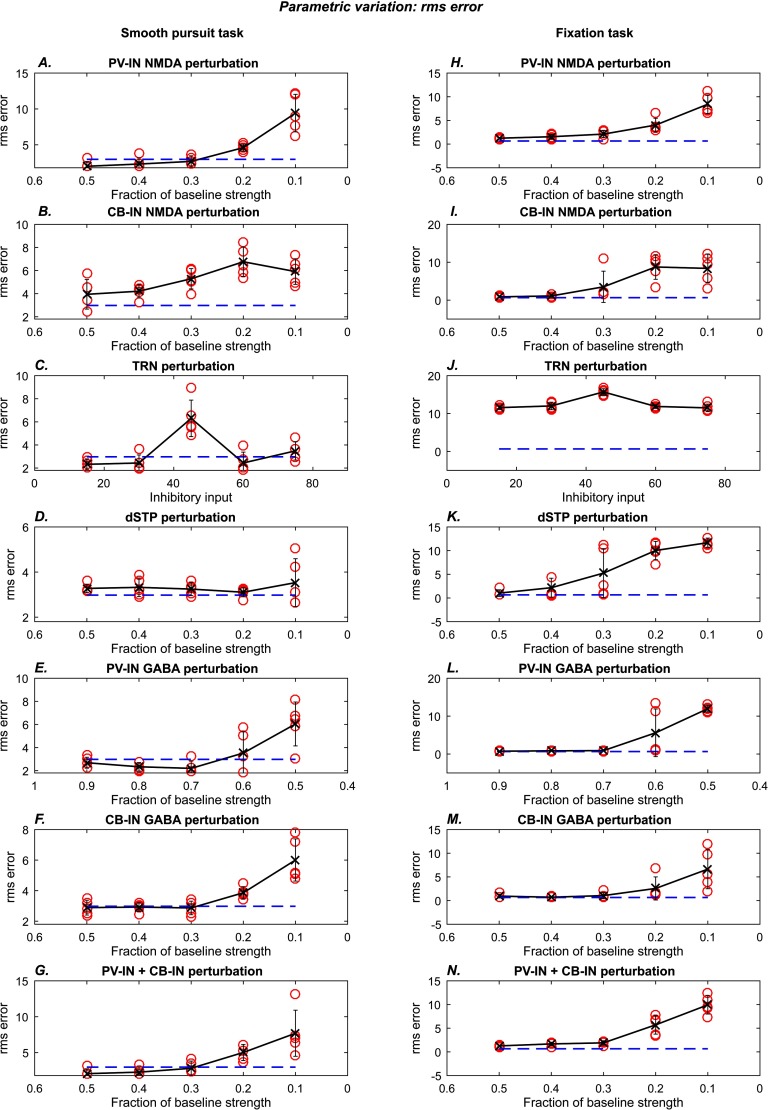
**Error as a function of perturbation strength.** Each subplot shows the root mean square (rms) error as a function of the strength of the simulated perturbation. The blue dashed lines show the average rms error for the normal performance epoch (4 to 6 s period). Red circles show the rms error for the perturbed performance epoch (6 to 8 s period) for a single trial. The black lines shows the trend of the averages across five simulation trials for a given perturbation level. Error bars indicate standard deviation. Plots on the left show results for the smooth pursuit task. Plots on the right show results for the fixation task.

**Figure 10.**
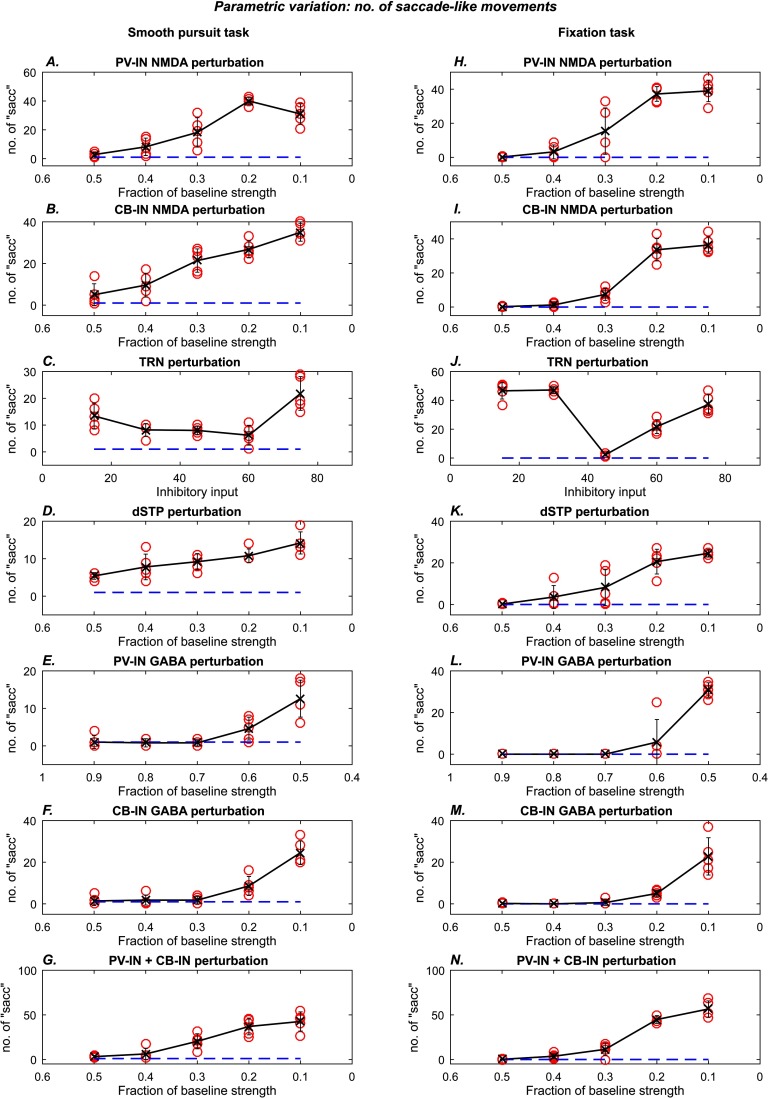
**Number of “saccade-like” transitions as a function of perturbation strength.** Each subplot shows the number of high-velocity “saccade-like” movements as a function of the strength of the simulated perturbation. The blue dashed lines show the average “saccade” number for the normal performance epoch (4 to 6 s period). Red circles show the “saccade” number for the perturbed performance epoch (6 to 8 s period) for a single trial. The black lines show the trend of the averages across five simulation trials for a given perturbation level. Error bars indicate standard deviation. Plots on the left show results for the smooth pursuit task. Plots on the right show results for the fixation task.

We simulated the effect of simultaneous NMDA disruption for both PV-INs and CB-INs. The PV-IN perturbation disinhibited MLNs, while the CB-IN perturbation caused increased recruitment of MLNs. Thus the oscillatory errors associated with the PV-IN perturbation occurred alongside the increased alpha power associated with the CB-IN perturbation. We also simulated direct reduction of the inhibitory strength of PV- and CB-INs. The performance deficits were broadly similar to the NMDA-based perturbations described earlier, particularly in the case of the direct CB-IN manipulation. The direct PV-IN perturbation caused more erratic eye movements than the NMDA-based perturbation for the parameter ranges used in the simulations shown here. Performance statistics for these additional perturbations are shown in Figures 9 and 10.

#### Quantitative Analysis of Model Performance and Perturbations

We systematically increased the magnitude of each perturbation to demonstrate the evolution of abnormal performance. We used the following performance measures: root mean square (rms) eye position error (Figure 9) and number of high-velocity (saccade-like) eye movements (Figure 10). Temporal locations of the high-velocity (saccade-like) eye movements are depicted with vertical black ticks in Figure 7. Compared to healthy controls, schizophrenia patients tend to show greater rms error in smooth pursuit tasks (Allen et al., 1990; Benson et al., 2012; O’Driscoll & Callahan, 2008; Ross et al., 1997) and a larger number of catch-up and intrusive saccades in fixation tasks (Benson et al., 2012; Litman et al., 1997; Rybakowski & Borkowska, 2002). We computed performance measures for the normal epoch (4–6 s period) and the perturbed epoch (6–8 s period).

For the PV-IN (Figures 9A and 9H; Figures 10A and 10H) and CB-IN (Figures 9B and 9I; Figures 10B and 10I) perturbations, we decreased the NMDA conductance in increments of 10%, starting from the 50% point. For the TRN perturbation (Figures 9C and 9J; Figures 10C and 10J), we increased the hyperpolarizing input in steps of 15 units. For the dSTP perturbation (Figures 9D and 9K; Figures 10D and 10K), we decreased the strength of disinhibition in steps of 10%, starting from the 50% point. We also simulated direct reductions in the strength of the GABAergic inhibition from the PV-INs (Figures 9E and 9L; Figures 10E and 10L, ranging from 90% to 50%) and from the CB-INs (Figures 9F and 9M; Figures 10F and 10M, ranging from 50% to 10%). Last, we simulated the combined effect of reducing NMDA input on both PV-INs and CB-INs simultaneously (Figures 9G and 9N; Figures 10G and 10N). The perturbations synergistically contributed to reduced performance.

We performed the parametric exploration of each perturbation described above for five instances of the model. Each instance was initialized with distinct neural parameters and was subject to different noise patterns. The qualitative effects were robust. In Figures 9 and 10, blue dashed lines represent average baseline measures from the unperturbed epoch, and red circles represent measures from the perturbed epoch. We now describe the average trend (black line) observed from averaging the performance measures of five instances of the model.

All the simulated perturbations were capable of increasing rms error and number of high-velocity movements. For both tasks, the average number of high-velocity movements increased with increase in the degree of the perturbation, for all except the TRN manipulation, which is described further later. The average rms error rate also went up as a function of perturbation strength, but no trend was visible in the smooth pursuit task for the dSTP and TRN manipulations. The parametric plots in Figure 9 show that the ratio of rms errors in the perturbed epoch versus the normal epoch is comparable to the ratios observed in human subjects: The ratio of total rms errors in schizophrenia patients versus healthy controls varied across studies but fell between 1.1 and 1.9 (Allen et al., 1990; Benson et al., 2012; Ross et al., 1997), which is within the range observed in our simulations (Figure 9).

Increasing the TRN hyperpolarization had a nonmonotonic effect on the performance measures (Figures 9C and 9J; Figures 10C and 10J). This occurred because as hyperpolarization is increased, the TRN passes through a stage in which it is completely inactive, and selective filtering of thalamo-cortical signaling is completely absent. Beyond this parameter range, further hyperpolarization leads to opening of the T-type calcium channels, which enables the TRN to become active once again and partially restore tracking performance. Thus the nonlinearity of the dynamics of T-type calcium channels leads to a nonlinear effect of hyperpolarizing TRN neurons.

## DISCUSSION

Schizophrenia is a severe disorder that manifests as a complex set of perceptual, emotional, and cognitive abnormalities. Among cognitive deficits, disruptions of attention-related eye movements are prominent and serve to distinguish schizophrenia patients from healthy controls (Benson et al., 2012). Our computational model of visual stimulus pursuit and fixation demonstrates how specific neural perturbations can trigger schizophrenia-like eye movement dysfunctions. Our simulations focused on disrupting inhibition, which has been linked with schizophrenia in a variety of empirical studies (reviewed in Glausier & Lewis, 2017; Reynolds, Abdul-Monim, Neill, & Zhang, 2004). Schizophrenia has been associated with abnormalities in cortical parvalbumin (PV) interneurons (Bastrup & Larsen, 2017; Chung et al., 2016; Steullet et al., 2017), cortical calbindin (CB) interneurons (Beasley et al., 2002; Chance, Walker, & Crow, 2005; Woo et al., 2008), and the wholly inhibitory TRN, which inhibits the dorsal thalamus (Ferrarelli & Tononi, 2011; Pratt et al., 2017; Pratt & Morris, 2015). The CRT circuit is the minimal connectivity pattern that integrates these schizophrenia-related inhibitory mechanisms and was therefore the basis for our model. The CRT network motif recurs across the major sensory, association, and motor systems in the brain, so understanding its properties in both normal and disordered conditions has implications beyond the attention-related symptoms of schizophrenia.

Our simulations showed that the CRT circuit can mediate smooth pursuit of a moving stimulus and fixation on a stationary stimulus in the presence of distractors. Disordered pursuit and fixation, which are characteristic of schizophrenia, are triggered by perturbations of three crucial sources of inhibition in the circuit. Specifically, weakened inhibition from (a) cortical PV interneurons that provide perisomatic inhibition of pyramidal neurons, (b) cortical CB interneurons that inhibit distal dendrites of pyramidal neurons, or (c) TRN neurons that inhibit thalamo-cortical neurons can yield deficits in stimulus tracking and fixation. Disruption of the cortex-regulated short-term depression of TRN inhibition (Crandall et al., 2015) also degraded performance on these tasks.

We modeled the dysfunction of cortical interneurons through downregulation of excitatory NMDA receptors. Dysfunction of NMDA receptors in schizophrenia is central to the glutamatergic hypothesis of schizophrenia (Alherz et al., 2017; Kehrer et al., 2008; Olney et al., 1999) as well as the disconnection hypothesis (Friston, 1998; Stephan, Friston, & Frith, 2009). There is evidence that the NMDA receptors on GABAergic neurons are disrupted in schizophrenia (Coyle, 2004; Olney & Farber, 1995). The model suggests a plausible mechanism through which NMDA dysfunction on cortical interneurons can contribute to schizophrenia-like symptoms through reduction in GABAergic inhibition onto cortical pyramidal neurons. Preliminary simulations (not shown) indicated that direct reduction of GABA strength and reduction of NMDA conductance into PV and CB interneurons have analogous and broadly similar effects, consistent with findings in schizophrenia patients (Glausier & Lewis, 2017). The diversity of effects that yield weakened inhibition in schizophrenia is also consistent with the interindividual variability of the disease and the specific insults that trigger symptoms.

Our simulations suggest that any reduction in inhibition of cortical pyramidal neurons can have similar effects on stimulus tracking. Significantly, each type of simulated perturbation has distinctive behavioral features, consistent with the proposal that schizophrenia is not one disorder but a spectrum of related disorders that can arise through multiple neural dysfunctions (Clementz et al., 2015). These behavioral differences were accompanied by differences in neural dynamics: Only the CB-interneuron and dSTP perturbations produced sustained increases in alpha power (∼10 Hz) in both tasks. Brain rhythms in the alpha range have been linked with active attentional suppression and sleep (Cantero, Atienza, & Salas, 2002; Foxe & Snyder, 2011; Vijayan & Kopell, 2012) and are altered in schizophrenia (Hong, Summerfelt, Mitchell, O’Donnell, & Thaker, 2012; Pratt et al., 2017; Uhlhaas, Haenschel, Nikolić, & Singer, 2008).

Our model thus supports the idea that distinct mechanistic disruptions to the circuit may result in different subtypes of schizophrenia. Experimental studies suggest that brain-based biomarkers can be used to identify broad psychosis subtypes (Clementz et al., 2015). Computational models may help refine and focus these subtypes by suggesting candidate biomarkers and activity “signatures” for future experimental studies. Once such correlates are reliably mapped to symptoms, computational models can be used to explore personalized therapies.

The simulated neural perturbations that disrupt stimulus tracking may have analogues in parallel CRT circuits devoted to very different cognitive, attentional, and perceptual processes. Similar perturbations in functionally distinct CRT circuits may lead to other schizophrenia-related symptoms. One of these pertains to the known deficit in working memory in schizo phrenia (Murray et al., 2014), which likely includes a cortico-thalamic loop through lateral prefrontal cortex and the thalamic mediodorsal nucleus. Interestingly, some lateral prefrontal areas have extended connections with the TRN (Zikopoulos & Barbas, 2006), and their disruption in schizophrenia likely affects the corresponding CRT circuits.

### Linking “Selective Disinhibition” to Other Symptoms of Schizophrenia

Selective disinhibition, a novel computational mechanism derived from a recent experimental finding (Crandall et al., 2015), may be relevant to multiple symptoms of schizophrenia. Our model appears to be the first to incorporate this mechanism, through which persistent elevated firing in deep cortical neurons weakens the effect of TRN inhibition on the corresponding thalamic neurons and thereby reduces feedback (or “closed-loop”) inhibition. In the absence of stable cortical firing, thalamo-cortical signaling remains in an “intermittent attention” mode, in which closed-loop inhibition prevents any CRT loop from becoming strong enough to suppress competitors. In the sensory processing domain, this amounts to a distractible state in which attention is spread broadly, thinly, and inefficiently. Thus the ability to regulate the stability of firing in deep cortical neurons may be crucial to several processes that depend on thalamo-cortical signaling.

Unstable cortical representations may contribute to various symptoms of schizophrenia. For example, disordered or irrelevant thoughts may arise if the relevant CRT circuits cannot maintain a stable representation of the current context. In such situations, context-appropriate signals may be weakly transmitted from thalamus to cortex. In parallel, context-inappropriate thoughts or actions, represented by competing CRT loops, may fail to be inhibited adequately, just as the distractor fails to be inhibited in the attentional fixation task simulated here. The top-down signal from the deep cortical layers to the thalamus may therefore play a powerful role in modulating the signal-to-noise ratio of thalamo-cortical signaling. Such a framework may provide mechanistic insight into the weakened ability of schizophrenia patients to inhibit contextually irrelevant information (Titone, Levy, & Holzman, 2016). Disrupted contextual processing has been identified as a unifying framework for understanding schizophrenia (Hemsley, 2005).

### Relationship of the Model to the Wider Visuomotor Network

Our model implements stimulus tracking and fixation behavior by folding together several distributed visuomotor processes into a unified CRT circuit, enabling a degree of generalizability beyond eye movements and visual attention. Simulating a richer and more realistic repertoire of visuomotor behaviors, which may require distinct mechanisms for target recognition, attention enhancement, fixations, saccades, and smooth pursuit, necessitates a complex distributed network containing multiple thalamo-cortical stages, as illustrated by other computational models (e.g., Srihasam et al., 2009). This network includes cortical regions—the FEF, the supplementary eye field (SEF), lateral/medial intraparietal cortices (LIP/MIP), middle temporal cortex, and middle superior temporal cortex (MST)—and associated subcortical regions, including the linked thalamic sectors and SC (Krauzlis et al., 2013; Rao et al., 2016; Schall, 2015; Smith et al., 2009; Tian & Lynch, 1997).

Several lines of evidence suggest that the functions mediated by our model CRT circuit can be mapped onto the CRT loop linking FEF, TRN, and the mediodorsal thalamus (MD). The model’s cortical stage is the source of motor commands that redirect the eyes, a function long identified with the FEF (reviewed in Schall, 2015). Additional signals for the genesis of oculomotor commands are generated by LIP/MIP and SEF, but neither area aligns as well as the FEF, which is organized as a retinotopic map of a target’s retinal eccentricity. Neither LIP/MIP nor SEF neurons have stable retinotopic receptive fields, while about 60% of FEF neurons do (Caruso et al., 2018). Furthermore, FEF is the most vital node for voluntary selective attention, because like the SC (and unlike LIP), its activation is directly under the control of thalamic zones gated by basal ganglia decisions. Unlike the SC, and like other premotor cortices, activation of FEF reflects not only eye movement commands but also learned cueing or suppression of those commands in paradigms that require selective attention (review and model in Brown, Bullock, & Grossberg, 2004). If the modeled cortical stage corresponds to FEF, then lateral MD, often called the *oculomotor thalamus* (Hulme, Whiteley, & Shipp, 2010), corresponds to the modeled thalamic nucleus given its connectivity with FEF (Barbas & Mesulam, 1981; Jones, 2007; Xiao, Zikopoulos, & Barbas, 2009).

Future extensions of our model can address many more schizophrenia symptoms by linking the FEF with other areas. For example, the FEF normally synchronizes with LIP just prior to saccades (Buschman & Miller, 2007), a phenomenon that may be mediated by a thalamic relay (Gollo, Mirasso, & Villa, 2010; Viriyopase, Bojak, Zeitler, & Gielen, 2012), and is deficient in many schizophrenic patients (Gonzalez-Burgos, Cho, & Lewis, 2015; Hirvonen et al., 2017; Krishna, O’Neill, Sánchez-Morla, & Thaker, 2014; Uhlhaas & Singer, 2011). In addition, FEF exhibits hypoconnectivity with the central thalamus, striatum, and cerebellum in schizophrenia patients. Anticevic et al. (2015) recently reported that individuals at high risk of conversion to schizophrenia were more likely to convert if they exhibited more extreme hypoconnectivity among these nodes.

The oculomotor regions discussed herein also participate in predictive signals, which have not been incorporated here because only retinal eccentricity is used to guide eye movements, and the model does not predict the future stimulus position. Eye movement tasks that do not involve prediction are disrupted in schizophrenia, including smooth pursuit of unpredictable curves, such as lissajous curves (i.e., curves that exhibit complex two-dimensional harmonic motion; e.g., Allen et al., 1990; Benson et al., 2012).

### Comparisons With Human Data

Our primary goal was to show how the modeled disruptions can affect performance mediated by a model CRT circuit rather than provide close fits of human eye movement data, which is beyond the scope of the present study. Nevertheless, the number of degrees of freedom in the model facilitates quantitative fits in performance measurements, as illustrated in Figures 9 and 10. The range of ratios of the rms errors in the perturbed epoch versus the normal epoch (Figure 9) is comparable with the range reported in the literature, which varied from 1.1 to 1.9 (Allen et al., 1990; Benson et al., 2012; Ross et al., 1997).

Furthermore, while many studies agree on the presence of eye movement disruptions in schizophrenia, there is inconsistency at the quantitative level, with some studies reporting significant changes in eye movement gain (e.g., Litman et al., 1997; Ross et al., 1997), while others report either weak or absent effects (e.g., Allen et al., 1990; Benson et al., 2012). Such discrepancies appear to arise from differences in task structure and data analysis methods, and possibly also from variability in the schizophrenia patient population (e.g., Nkam et al., 2010).

### Comparison With Other Schizophrenia-Related Models

To our knowledge, this is the first computational model of stimulus tracking in schizophrenia that uses a spiking neuron framework and also the first to demonstrate specific symptoms arising from dysfunction in distinct classes of cortical and TRN inhibitory neurons. Other approaches to modeling eye movements in schizophrenia involve concepts such as predictive coding and active inference (Adams, Perrinet, & Friston, 2012; Heinzle, Aponte, & Stephan, 2016). These learning theory–based approaches operate on a more abstract level than spiking neuron models, but they are consistent with the neuronal mechanisms simulated here.

Another computational model of schizophrenia focused on the effects of dopamine on prefrontal contributions to cognitive tasks but is also more abstract than our model, as it does not specify detailed neuronal mechanisms, such as GABA or NMDA action (Braver, Barch, & Cohen, 1999). Our model is complementary to the more abstract models of stimulus tracking and therefore does not contradict them. Further research may suggest ways to combine the best features of both modeling approaches. For example, it may be productive to implement parts of the predictive coding component of abstract models using the neural mechanism for top-down influence simulated here: selective disinhibition. Specifically, our model points to a crucial role for deep cortical neurons: control of selective disinhibition of thalamic projecting neurons, which mediates top-down representations of stimulus position and is therefore consistent with the active inference model (Adams et al., 2012). Predictive cortical representations, not modeled here, may use this disinhibition mechanism to mediate forms of top-down expectation.

Prior modeling work has investigated aspects of the role of NMDA in abnormalities of working memory in schizophrenia, but stimulus tracking was not directly addressed (Krystal et al., 2017; Murray et al., 2014). The latter work showed that reducing lateral inhibition from interneurons can affect tuning of cortical ensembles associated with working memory. Our findings are consistent with this effect. Our simulations show that abnormally broad tuning, arising from weak inhibition either in cortex or thalamus, results in erratic tracking and susceptibility to distractors.

The model presented here employs the simplest connectivity pattern capable of linking the GABAergic mechanisms in cortex and thalamus associated with schizophrenia. As a biophysical model, the level of complexity is higher than in models focused on minimizing the number of parameters required to fit behavioral data. The model occupies a position between phenomenological nonneural models and detailed biophysical network models with multiple cortical and subcortical structures.

### Conclusion

The computational model presented here demonstrates plausible neurobiological mechanisms for stimulus tracking and fixation and shows how localized disruptions can produce schizophrenia-like symptoms. The CRT circuit modeled here applies broadly beyond oculomotor behavior, because it is a motif that recurs for all sensory, association, and motor systems and serves to integrate these systems. Future work modeling interactions among multiple CRT circuits may allow us to investigate how localized disruptions simulated here can impact global brain connectivity and dynamics that are altered in schizophrenia (Anticevic et al., 2015; Tu, Hsieh, Li, Bai, & Su, 2012).

## METHODS

### Spiking Model

Six neuron groups were simulated: thalamus (T), TRN (R), middle-layer cortical pyramidal neurons (M), deep-layer cortical pyramidal neurons (D), cortical parvalbumin interneurons (PV), and cortical calbindin interneurons (CB). Each group of cortical pyramidal neurons had 120 neurons. The remaining groups each had 40 neurons.

The model is composed of spiking Izhikevich neurons (Izhikevich, 2003). Each model neuron is governed by the coupled differential equations

$$\tau_{s}\frac{dv}{dt}=0.04v^{2}+5v+140-u+\Sigma I$$ (1)

$$\tau_{s}\frac{du}{dt}=a\left(bv-u\right),$$ (2)

along with an auxiliary after-spike resetting:

$$\text{if}\;v\geq 30,\text{then}\left\{\begin{array}{l} v\leftarrow c\\ u\leftarrow u+d, \end{array}\right.$$ (3)

where *v* represents the voltage of the neuron (in millivolts), *u* is a recovery variable, and *a*, *b*, *c*, and *d* are parameters that facilitate approximating various types of spiking neuron (Izhikevich, 2003). The integration time constant is *τ*_*s*_. The term Σ*I* is the total input current. Neural sources of excitatory and inhibitory inputs to each neuron group are depicted in Figure 1. The excitatory and inhibitory currents contributing to Σ*I* are listed in Table 1, which follows their individual specifications.

**Table 1.** Inputs to each neuron group

**Region**	**Excitatory currents**	**Inhibitory currents**
Thalamus (T)	*I*_ext_^AMPA^ + *I*_ext_^NMDA^ + *I*_*D*→*T*_^AMPA^ + *I*_*T*_^Ca^	*P*_*T*_ *I*_*R*→*T*_^GABA_*B*_^
TRN (R)	*I*_*T*→*R*_^AMPA^ + *I*_*D*→*R*_^AMPA^ + *I*_*R*_^Ca^ + *I*_*R*→*R*_^gap^	*I*_hyp_
Middle-layer pyramidal neurons (M)	*I*_*T*→*M*_^AMPA^ (*)	*I*_PV→*M*_^GABA_*A*_^
Middle-layer PV neurons (PV)	*I*_*M*→PV_^AMPA^ + *I*_*M*→PV_^NMDA^	*I*_PV→PV_^GABA_*A*_^
Middle-layer CB neurons (CB)	*I*_*M*→CB_^AMPA^ + *I*_*M*→CB_^NMDA^	0
Deep-layer pyramidal neurons (D)	*I*_*M*→*D*_^AMPA^	0

For the majority of model neurons, the values of *a*, *b*, *c*, and *d* in Equations 1–3 are chosen to simulate a regular spiking neuron type (0.02, 0.2, −65, and 0, respectively). All PV inhibitory neurons have values of *a*, *b*, *c*, and *d* chosen to simulate a fast-spiking neuron type (0.1, 0.2, −65, and 2, respectively).

### Glutamatergic and GABAergic Currents

To model the postsynaptic conductances that control synaptic currents, we use the saturating differentials (SD) spike-dependent signal *g*_SD_ (Palma, Grossberg, & Versace, 2012). Thus all postsynaptic conductances are governed by a couplet of differential equations of the form

$$\tau_{s}\frac{dQ}{dt}=\left(1-Q\right)K-\frac{Q}{\tau_{\text{rise}}}$$ (4)

$$\tau_{s}\frac{dg_{\text{SD}}}{dt}=\left(\frac{\tau_{\text{fall}}+\tau_{\text{rise}}}{\tau_{\text{fall}}}\right)\left[\frac{2}{\tau_{\text{rise}}}\left(1-g_{\text{SD}}\right)Q-\frac{g_{\text{SD}}}{\tau_{\text{fall}}}\right].$$ (5)

For each synapse type, an intermediate variable *Q* is triggered by the arrival of a discrete spike *K* and can be interpreted as the presence of transmitter in the synaptic cleft. The spike variable *K* takes the value of 1 at the moment the voltage *v* goes above a threshold (30 mV), then resets to zero. Dynamics of different types of synapses are captured by setting values of parameters *τ*_rise_ and *τ*_fall_, which determine the rise and fall times, respectively, of the postsynaptic conductances. These values for each neuron group, and that of parameter *τ*_*s*_, the time constant of integration, are listed in Table 2. Overall, the dynamics of the *g*_SD_ approximate the short- and medium-term components of postsynaptic conductances. This formulation (adopted to benefit from the computational speed-up offered by the Izhikevich approximations compared with Hodgkin–Huxley formulations for spiking neurons) means that the fast temporal dynamics of the variable *Q* and the after-spike resetting are not independent of the choice of integration time step. To preserve results across time step settings, changes of time step can be compensated by appropriate changes to the duration and magnitude of *K*.

**Table 2.** Model parameters

**Term**	**Value**
*σ*_*D*→*T*_	0.05
*σ*_*T*→*M*_^*p*^	0.0625
*σ*_*T*→*M*_^*d*^	0.9375
*σ*_*M*→*D*_	0.0125
*σ*_*T*→*R*_	0.0938
*σ*_*D*→*R*_	0.0125
*σ*_*R*→*R*_^gap^	0.0313
*σ*_PV→PV_	0.0313
*σ*_PV→*M*_	0.4375
*σ*_CB→*M*_	1.25
*σ*_*M*→PV_	0.025
*σ*_*M*→CB_	0.125
*τ*_rise_^fast^	2 ms
*τ*_fall_^fast^	10 ms
*τ*_rise_^NMDA^	8 ms
*τ*_fall_^NMDA^	100 ms
*τ*_rise_^PV^	0.1 ms
*τ*_fall_^PV^	0.5 ms
*τ*_rise_^GABA_*B*_^	8 ms
*τ*_fall_^GABA_*B*_^	30 ms
*τ*_rise_^xslow^	200 ms
*τ*_fall_^xslow^	400 ms
*τ*_*s*_	0.001 ms
*A*_*D*→*T*_	0.04
*A*_*T*→*M*_^*p*^	4.8
*A*_*T*→*M*_^*d*^	1.2
*A*_*M*→*D*_	1.4
*A*_*T*→*R*_	0.15
*A*_*D*→*R*_	1
*A*_*R*→*R*_^gap^	0.3
*A*_PV→PV_	10
*A*_PV→*M*_	50
*A*_CB→*M*_	1
*A*_*M*→PV_^AMPA^	0.5
*A*_*M*→PV_^NMDA^	4
*A*_*M*→CB_^AMPA^	0.03
*A*_*M*→CB_^NMDA^	1
*A*_*R*→*T*_	0.32
*A*_*T*_^Ca^	20
*A*_*R*_^Ca^	12
*A*^STP^	3,000
*N*_*T*_, *N*_PV_, *N*_CB_, *N*_*R*_	40
*N*_*M*_, *N*_*D*_	120

Synaptic currents depend on summed conductances and the postsynaptic voltage. The AMPA current inputs *I*_pre→post_^AMPA^ to a postynaptic neuron are computed as follows:

$$I_{\text{pre}\to \text{post}}^{\text{AMPA}}=A_{\text{pre}\to \text{post}}^{\text{AMPA}}v_{\text{post}}\underset{\text{pre}}{\sum }W_{\text{pre}\to \text{post}}g_{\text{pre}}^{\text{fast}},$$ (6)

where post and pre represent the indices of the postsynaptic and presynaptic neurons, respectively, *A*_pre→post_^AMPA^ is an amplitude, and the sum is taken over conductances reflecting weighted inputs from all presynaptic neurons. Similarly, the NMDA current inputs are computed as follows:

$$I_{\text{pre}\to \text{post}}^{\text{NMDA}}=A_{\text{pre}\to \text{post}}^{\text{NMDA}}v_{\text{post}}B\left(v_{\text{post}}\right)\underset{\text{pre}}{\sum }W_{\text{pre}\to \text{post}}g_{\text{pre}}^{\text{NMDA}},$$ (7)

where *A*_pre→post_^NMDA^ is an amplitude. Both *A*_pre→post_^AMPA^ and *A*_pre→post_^NMDA^ take the value 1, unless specified otherwise in Table 2. For the inputs to the PV- and CB-INs, these amplitudes are set to make the qualitative effects of the NMDA perturbations clear. Also, there is a higher density of NMDA receptors in upper layers of the relevant cortical regions, where CB-INs are located (Palomero-Gallagher & Zilles, 2017).

The NMDA voltage dependence is determined by *B* (*v*) = [1 + 0.4202 exp (0.062 * *v*)]^−1^. Inhibitory GABAergic currents are given by the following equations:

$$I_{\text{pre}\to \text{post}}^{{\text{GABA}}_{A}}=\left(v_{\text{post}}+70\right)\underset{\text{pre}}{\sum }W_{\text{pre}\to \text{post}}g_{\text{pre}}^{\text{fast}}$$ (8)

$$I_{\text{pre}\to \text{post}}^{{\text{GABA}}_{B}}=\left(v_{\text{post}}+90\right)\underset{\text{pre}}{\sum }W_{\text{pre}\to \text{post}}g_{\text{pre}}^{{\text{GABA}}_{B}}.$$ (9)

In each of Equations 8 and 9, the connection weight matrix *W*_pre→post_ is different for each pair of connected regions. The synaptic events triggered by the PV neurons are faster than those of the other neuron groups, so the PV neuron time constants are specified separately in Table 1 (*τ*_rise_^PV^, *τ*_fall_^PV^).

### Gap Junction Coupling

Neurons in the TRN have short-range gap junction coupling:

$$I_{\text{pre}\to \text{post}}^{\text{gap}}=\underset{\text{pre}}{\sum }W_{\text{pre}\to \text{post}}^{\text{gap}}\left(v_{\text{pre}}-v_{\text{post}}\right).$$ (10)

### Calcium Current

To model thalamic oscillatory phenomena, we incorporate T-type calcium channels (Vijayan & Kopell, 2012; Zhan, Cox, Rinzel, & Sherman, 1999). The current produced in a neuron by T-type calcium channels is given by the following equation:

$$I_{\text{post}}^{\text{Ca}}=A_{\text{post}}^{\text{Ca}}P_{\text{Ca}}m^{2}h\frac{Vz^{2}F^{2}}{RT}\left[\frac{{\text{Ca}}_{\text{int}}-{\text{Ca}}_{\text{ext}}\exp\left(\frac{-zFV}{RT}\right)}{1-\exp\left(\frac{-zFV}{RT}\right)}\right],$$ (11)

where *A*_post_^Ca^ is an amplitude; *V* is the membrane potential (in volts); *m* and *h* are the activation and inactivation gates, respectively; *P*_Ca_ is the maximum permeability of an open channel (30 cm^3^/s); Ca_int_ and Ca_ext_ are the internal and external concentrations of calcium ions (the latter is assumed to be 2 mM); *z* is the charge of a calcium ion (+2); and *F*, *R*, and *T* are Faraday’s constant, the gas constant, and temperature in kelvins (298 K), respectively. At *v* = 0, *I*_post_^Ca^ = *A*_post_^Ca^*P*_Ca_*zF* (Ca_int_ − Ca_ext_).

Internal calcium concentration is governed by the following equation:

$$\frac{d{\text{Ca}}_{\text{int}}}{dt}={\left[\frac{-10}{2^{.}96485.3}I_{\text{Ca}}\right]}^{+}-0.2\left({\text{Ca}}_{\text{int}}-0.00024\right),$$ (12)

where the notation [*a*]^+^ represents a rectified linear transform of *a*.

The activation and inactivation gates are each governed by the following equation:

$$\frac{dx}{dt}=\frac{\left(x_{\infty }\left(v\right)-x\right)}{\tau_{x}\left(v\right)},$$ (13)

where *x* represents either *m* or *h*. The voltage-dependent terms are specified as

$$m_{\infty }\left(v\right)=\frac{1}{1+\exp\left(\frac{-\left(v+79\right)}{6.2}\right)},$$ (14)

$$h_{\infty }\left(v\right)=\frac{1}{1+\exp\left(\frac{v+92}{4}\right)},$$ (15)

$$\tau_{m}\left(v\right)=0.999+\frac{0.333}{\left(\exp\left(\frac{v+31}{10}\right)+\exp\left(\frac{-\left(v+106\right)}{15}\right)\right)},$$ (16)

$$\tau_{h}\left(v\right)=\frac{1}{3.737}\left(30.8+\frac{\left(211.4+\exp\frac{\left(v+119.2\right)}{5}\right)}{\left(1+\exp\frac{\left(v+90\right)}{3.2}\right)}\right).$$ (17)

To deinactivate the T-type calcium channels during perturbation epochs, we introduce a direct hyperpolarizing input *I*_hyp_.

### Short-Term Plasticity of TRN Synapses

A key feature of the model is the short-term depression of inhibitory TRN synapses on thalamic neurons (T), which is governed by activity in the deep or infragranular cortical pyramidal neurons (D). This plasticity is modeled phenomenologically, as a multiplicative modulatory factor *P*_*T*_*i*__ for each thalamic neuron, given by

$$P_{T_{i}}={\left[1+A^{\text{STP}}\underset{j}{\sum }W_{D_{j}\to T_{i}}{\left(g_{D_{j}}^{\text{xslow}}\right)}^{6}\right]}^{-1},$$ (18)

where *g*_*D*_^xslow^ is an “extra slow” synaptic event modeled using Equations 4 and 5, *A*^STP^ is a scaling constant, and the sum is taken over all presynaptic neurons. *P*_*T*_ is bounded above by 1 and decreases toward zero as the activity of the presynaptic deep cortical neurons increases. In this way, the cortex can regulate the degree of inhibition on thalamic neurons. The amplitude *A*^STP^, the time constants of *g*_*D*_*j*__^xslow^, and the exponent of 6 in Equation 18 were selected so that *P*_*T*_*i*__ could approximate the temporal dynamics of the known plasticity. Preliminary simulations (not shown) indicated that other mechanisms can produce qualitatively similar disinhibition, including (a) a threshold on the term *g*_*D*_^xslow^ in Equation 18 and no exponent and (b) direct inhibition of the TRN inhibition on thalamic neurons, mediated by an implicit local interneuron.

### Connectivity

The asterisk in Table 1 indicates that the effect of CB neurons on middle-layer pyramidal neurons is not fully specified in the table. This effect is shown in the expanded form of the inputs to M:

$$I_{T\to M}^{\text{AMPA}}=A_{T\to M}^{p\text{AMPA}}v_{S}\underset{T}{\sum }W_{T\to M}^{p}g_{T}^{\text{AMPA}}+A_{T\to M}^{d\text{AMPA}}v_{S}{\left[\underset{T}{\sum }W_{T\to M}^{d}g_{T}^{\text{AMPA}}-\underset{\text{CB}}{\sum }W_{\text{CB}\to M}g_{\text{CB}}^{\text{fast}}\right]}^{+}.$$ (19)

Of the two products being added on the right-hand side, the first represents the net input to the proximal dendrites of the middle-layer neuron and the second represents the net input to the distal dendrites. Only the distal dendrites undergo inhibition from CB neurons. This inhibition cannot affect excitation on the proximal dendrites, so its term is rectified (denoted by [.]^+^) to prevent it from becoming negative. Weight matrix *W*_*T*→*M*_^*p*^ represents the connectivity between thalamic neurons (T) and the proximal dendrites of MLNs (M). Weight matrix *W*_*T*→*M*_^*d*^ represents the connectivity between thalamic neurons (T) and the distal dendrites of MLNs (M). This approach is a way to approximate differences between proximal and distal inputs without the additional computational complexity of compartmental modeling. *A*_*T*→*M*_^*p*AMPA^ and *A*_*T*→*M*_^*d*AMPA^ are amplitudes.

In these equations and in Table 1, the indices of individual neurons are omitted to enhance readability. Letters in subscripts represent region names rather than specific neurons. This notation can be expanded to specify the indexes of individual neurons. For example, consider the connection between the deep cortical neurons (D) and the thalamus (T). Let region *D* have *N*_*D*_ neurons and region *T* have *N*_*T*_ neurons. The cumulative AMPA current generated by all deep cortical neurons (D) synapsing on the *j*th thalamic neuron is given by

$$I_{D\to T_{j}}^{\text{AMPA}}=v_{T_{j}}\overset{N_{D}}{\underset{i=1}{\sum }}W_{D_{i}\to T_{j}}g_{D_{i}}^{\text{fast}},$$ (20)

where *W*_*D*_*i*_→*T*_*j*__ is the (*i*, *j*) element of the *N*_*T*_ ⋅ *N*_*D*_-dimensional connectivity matrix. Most of the model connection weights are distance dependent and determined by Gaussian curves. So the connectivity between deep cortical pyramidal neurons and thalamic neurons is given by

$$W_{D_{i}\to T_{j}}=A_{D\to T}\exp\left[-{\left(\frac{i/N_{D}-j/N_{T}}{\sigma_{D\to T}}\right)}^{2}\right].$$ (21)

The connection weights *W*_*M*_*i*_→*D*_*j*__, *W*_*T*_*i*_→*M*_*j*__^*p*^, *W*_*T*_*i*_→*M*_*j*__^*d*^, *W*_*M*_*i*_→PV_*j*__, *W*_*M*_*i*_→CB_*j*__, *W*_*T*_*i*_→*R*_*j*__, *W*_*D*_*i*_→*R*_*j*__, and *W*_*R*→*R*_^gap^ are also of Gaussian form, with parameters for width (*σ*_pre→post_) and amplitude (*A*_pre→post_) specified in Table 2. Table 2 also specifies the number of neurons in each population.

The connection from the TRN to the thalamus, *W*_*R*_*i*_→*T*_*j*__, is uniformly all-to-all, with each weight taking the value *A*_*R*→*T*_. “Islands” or “holes” in the inhibition pattern arise solely due to the short-term plasticity triggered by cortical influence on TRN-thalamus synapses.

### Eye Movement Signals

The movement of the model’s center of gaze is modeled phenomenologically. The middle cortical layer is treated as a motor error map, with one-half of the neurons (indices 1 to *N*_*M*_/2) triggering a leftward movement and the other half (indices *N*_*M*_/2 + 1 to *N*_*M*_) triggering rightward movement. Thus a leftward movement is given by

$$L=\overset{N_{M}/2}{\underset{i=1}{\sum }}W_{i}^{L}g_{M_{i}}^{\text{fast}}.$$ (22)

Similarly, a rightward movement is given by

$$R=\overset{N_{M}}{\underset{i=N_{M}/2+1}{\sum }}W_{i}^{R}g_{M_{i}}^{\text{fast}},$$ (23)

where the weights are given by

$$W_{i}^{L}=0.025{\left[\frac{0.5N_{M}-i}{0.5N_{M}}\right]}^{+}$$ (24)

and

$$W_{i}^{R}=0.025{\left[\frac{i-0.5N_{M}}{0.5N_{M}}\right]}^{+}.$$ (25)

The center of gaze is directly modulated by the two movement signals as follows:

$$x_{c}\left(t\right)=x_{c}\left(t-1\right)+R-L.$$ (26)

Thus, if the rightward signal and leftward signal are counterbalanced, the gaze is fixed at a particular location.

### External Inputs

Sensory inputs to the thalamus take the form of a Poisson series of spike events. The postsynaptic potentials triggered by these spikes are governed by Equations 4 and 5. Each stimulus and distractor is modeled as a Gaussian bump that controls the instantaneous Poisson rate of the input. The Poisson input rate of the *i*th thalamic input is governed by

$$S_{i}\left(t\right)=30+220\cdot \exp\left[-{\left(\frac{0.5N_{T}-i-(x_{c}\left(t\right)-x_{T}\left(t\right))}{4}\right)}^{2}\right],$$ (27)

where *x*_*T*_(*t*) is the center of the target. For the smooth tracking task, this is governed by a sinusoid that moves between the minimum (*i* = 11) and maximum (*i* = 30) positions at a frequency of once per second. The baseline Poisson rate is 30 spikes per second, and the maximal Poisson rate is 250 spikes per second. For the fixation task, *x*_*T*_(*t*) is a constant (*i* = 10), and the distractor is of equal width and amplitude, located to one side of the target (*i* = 25). In both tasks, eye position was bound between positions 5 and 36. In the case of very large abnormal eye movements, the model eye may reach these bounds.

### Simulation Details

All simulations were performed in MATLAB (R2015b). The model code is available at http://www.bu.edu/neural/Models/SchizModel.zip. The equations were integrated using the forward Euler method, with a step size *h* = 0.025 ms. For a given set of perturbations, the membrane potentials, spiking parameters (*a*, *b*, *c*, *d*), and synaptic rise and fall times of each neuron were initialized with variability within ±5% to ensure that the model neurons in each population were not exactly identical.

For the PV and CB perturbations, the corresponding NMDA amplitude (*A*_*M*→PV_^NMDA^ or *A*_*M*→CB_^NMDA^) was decreased to 10% of the normal level in the final 2 s. For the TRN perturbation, an additional hyperpolarizing input current (*I*_hyp_) of −60 was introduced in the final 2 s. For the STP perturbation, the corresponding amplitude (*A*^STP^) was reduced to 10% of the normal level in the final 2 s.

For the parametric exploration of the PV-IN and CB-IN perturbations, we decreased the corresponding NMDA amplitude (*A*_*M*→PV_^NMDA^ and *A*_*M*→CB_^NMDA^, respectively) to 50%, 40%, 30%, 20%, and 10% of the normal level. For the TRN perturbation, we used the following hyperpolarizing currents (*I*_hyp_): −15, −30, −45, −60, −75. For the parametric exploration of the STP perturbation, we decreased the corresponding amplitude (*A*^STP^) to 50%, 40%, 30%, 20%, and 10% of the normal level. For the direct reduction of PV-IN GABA strength, we reduced the corresponding weights (*A*_PV→PV_ and *A*_PV→*M*_) to 90%, 80%, 70%, 60%, and 50% of the normal level. For the direct reduction of CB-IN GABA strength, we reduced the corresponding weight (*A*_CB→*M*_) to 50%, 40%, 30%, 20%, and 10% of the normal level. For the combined PV-IN and CB-IN perturbation, NMDA amplitudes for both types of IN (*A*_*M*→PV_^NMDA^ and *A*_*M*→CB_^NMDA^) were reduced together in steps of 50%, 40%, 30%, 20%, and 10% of the normal level.

The simulations were set up to show qualitatively what happens when the GABAergic disruptions are severe enough to create a functional difference between a “healthy” state and a perturbed state. To ensure low-error performance in the unperturbed mode, our main concern was to put the model in a region of parameter space that was well away from the error-producing zone. Had we specified the “healthy,” that is, low-error, model with parameters that were closer to the edge of failure (but still low error), then the changes needed to create functional deficits would have been much less than 50%. In a detailed model featuring multiple areas, and even with the initial parameters we used here to specify a “healthy” state, it is plausible that small disruptions of the type simulated here, acting in multiple reciprocally linked regions, or at multiple sites within the CRT circuits, could contribute synergistically to create a high error rate. More research will be needed to study these combinatoric issues.

### Data Analysis

We computed the spectrogram of middle-layer cortical activity as follows. The total activity (voltage) from all middle-layer pyramidal neurons was summed. The detrended sum was used as the input to the MATLAB (R2015b) “spectrogram” function. The sampling rate was 400 Hz. Window size for the spectrogram was 400 ms, and overlap between windows was 360 ms. We computed Fourier power spectra for two epochs—perturbed and normal—using the detrended total activity, with the MATLAB “fft” function. The perturbed epoch was the final 2 s of each trial, and the normal epoch was the preceding 2 s.

Root mean square (rms) error was computed as the square root of the mean squared difference between model eye position and target. The number of high-velocity movements was computed as the number of times the absolute instantaneous eye speed (in units of neuron number per sampling epoch of 2.5 ms) exceeded a threshold of 0.5. This measure counts large saccade-like movements as well as small, jerky movements. The number of degrees of retinal eccentricity represented by each neuron is a free-scaling factor in the model, so we left the units of rms error arbitrary.

## ACKNOWLEDGMENTS

This work was supported by grants from the National Institutes of Health: National Institute of Mental Health (R01MH057414, R01MH101209, MH117785), National Institute of Neurological Disorders and Stroke (R01NS024760), and the Center of Excellence for Learning in Education, Science and Technology (CELEST), a National Science Foundation Science of Learning Center (NSF OMA-0835976). The funders had no role in study design, data collection and analysis, decision to publish, or preparation of the manuscript.

## AUTHOR CONTRIBUTIONS

Conceptualization, Yohan J. John, Helen Barbas, Daniel Bullock, and Basilis Zikopoulos; Methodology, Yohan J. John; Formal Analysis, Yohan J. John; Software, Yohan J. John; Investigation, Yohan J. John, Helen Barbas, Daniel Bullock, and Basilis Zikopoulos; Writing—Original Draft, Yohan J. John; Writing—Review & Editing, Yohan J. John, Helen Barbas, Daniel Bullock, and Basilis Zikopoulos; Funding Acquisition, Helen Barbas; Supervision, Helen Barbas.
